# A Mobile Glycosylation Locus Modulates Cell Wall Architecture in *Lactobacillus crispatus*

**DOI:** 10.1111/mmi.70112

**Published:** 2026-09-10

**Authors:** P. Achterberg, M. Lekkerkerker, L. Koelemeijer, S. Illidge, J. Vroom, R. Y. Hertzberger, J. Vreede, M. C. M. van Loosdrecht, R. Kort

**Affiliations:** ^1^ Amsterdam Institute for Life and Environment (A‐LIFE) Vrije Universiteit Amsterdam Amsterdam the Netherlands; ^2^ Department of Biotechnology Delft University of Technology Delft the Netherlands; ^3^ Van't Hoff Institute for Molecular Sciences University of Amsterdam Amsterdam the Netherlands; ^4^ Wageningen Electron Microscopy Centre Wageningen University & Research Wageningen the Netherlands; ^5^ ARTIS‐Micropia Amsterdam the Netherlands

**Keywords:** genomic instability, glycosylation, glycosyltransferases, *Lactobacillus crispatus*, mobile genetic elements, vaginal microbiome

## Abstract

*Lactobacillus crispatus* dominance in the vaginal microbiome is associated with beneficial health outcomes, yet strain‐level variation and its implications remain poorly understood. Here, we resolve the genomic context of three glycosyltransferase gene fragments (GT1‐3) previously linked with dysbiotic states. Long‐read resequencing revealed that GT1‐3 are part of a ~18.7 kb Wzx/Wzy‐dependent cell wall polysaccharide (CWPS) locus, containing several IS256‐family transposases. Serial propagation in vitro produced isolates with 4.1 kb excised via a composite transposon encompassing the GT3, UDP‐galactopyranose mutase, flippase, and hypothetical protein, demonstrating structural plasticity. Transmission electron microscopy showed a ~20%–25% thinner peptidoglycan layer in the derived strains, while FT‐IR and monosaccharide analysis indicated no gross changes. Molecular dynamics simulations suggest that GT3 contributes to the structural stability of the glycosyltransferase complex, without compromising catalytic function. Together, these findings establish the CWPS locus as a mobile, structurally plastic element that directly influences cell wall architecture in *L. crispatus* .

## Graphical abstract

Serial propagation of *Lactobacillus crispatus* triggers transposon‐mediated loss of a mobile glycosylation gene fragment (GT3), destabilising the glycosyltransferase complex and remodelling the cell wall with 20%–25% thinner peptidoglycan, while sugar composition remains unchanged.

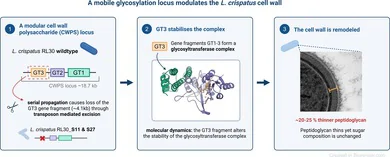

**Abbreviations**
CWPS locus: ~18.7  kb Wzx/Wzy‐dependent cell wall glycan biosynthesis locus
GT1‐3: glycosyltransferase fragments 1‐3 as previously described in Van der Veer et al. 2019
Internal mobile module: ~13 kb mobile part of CWPS locus harbouring the GT1‐3
Partial internal mobile module: 4.1 kb IS256‐flanked mobile element that is lost under serial propagation
UGM: UDP‐galactopyranose mutase (EC 5.4.99.9)
WT: wildtype strain

## 1 Introduction

The vaginal microbiome plays a critical role in women's reproductive health. A *Lactobacillus*‐dominated community, particularly enriched in *Lactobacillus crispatus*, is associated with favourable reproductive and sexual health outcomes, including low vaginal pH, reduced mucosal inflammation, and lower risks for bacterial vaginosis and preterm birth (France et al. 2022; Sillen et al. 2025; Hertzberger et al. 2025). Although these associations are well established, the microbial traits that mechanistically sustain vaginal homeostasis remain incompletely understood. While *L. crispatus* is considered protective, not all strains are functionally equivalent; isolates display marked genetic and phenotypic heterogeneity (Illidge et al. 2024; Van der Veer et al. 2019; Fontana et al. 2021; Hertzberger et al. 2022; Tarracchini et al. 2023; Nori et al. 2025; Santarelli et al. 2025; de Kreek et al. 2026). Strain‐level variation in glycosylation genes has been linked to the vaginal state and the host's health outcomes. Certain glycosyltransferase gene fragments (GT1‐3) were reported to occur more frequently in *L. crispatus* isolates from dysbiotic communities (Van der Veer et al. 2019). Similarly, a putative cell surface glycan gene cluster in *L. jensenii* is enriched in strains from women who delivered preterm (Nori et al. 2023).

In Lactobacillaceae, surface glycans are typically encoded by discrete biosynthesis gene clusters, of which the Wzx/Wzy‐dependent pathway is most common (Deo et al. 2019; Ascari and Morona 2025). Alterations at such loci can modify cell wall structure and, consequently, influence bacteriophage sensitivity and immune modulation (Theodorou et al. 2019; Croatti et al. 2026). In the vaginal niche specifically, *L. crispatus* S‐layer compounds modulate activation of host‐immune receptors (Decout et al. 2024), and peptidoglycan compounds of lactobacilli inhibit the herpes virus (Adams et al. 2025). Glycan synthesis clusters and other surface‐associated loci in Lactobacillaceae are frequently located within highly variable genomic regions enriched in mobile elements, facilitating surface diversification (Sybesma et al. 2013; Deo et al. 2019). Notably, serial propagation of *Lacticaseibacillus rhamnosus* led to transposon‐mediated loss of *spaCBC*, pilus genes involved in mucin adhesion, demonstrating the plasticity of surface‐associated loci under in vitro conditions (Sybesma et al. 2013).

Building on these insights, we extend the work of Van der Veer et al. (2019) by reconstructing the genomic context of the GT1‐3 using long‐read sequencing and demonstrating that the locus is structurally plastic. Serial propagation of a strain harbouring the complete locus yielded derivatives with a defined deletion encompassing GT3 and adjacent genes, enabling direct assessment of how partial locus loss affects cell wall architecture. Molecular dynamics simulations were used to explore further structural consequences of GT3 loss for the glycosyltransferase complex.

## 2 Results

### 2.1 A Gene Cluster Predicted To Be Involved in Transmembrane Glycosylation

To characterise the genomic organisation of the previously identified glycosyltransferase fragments (GT1‐3) (Van der Veer et al. 2019), we re‐sequenced a selection of *L. crispatus* strains using long‐read sequencing, enabling reconstruction of the GT fragments and their surrounding genomic context. *L. crispatus* RL30, which encodes all three GT fragments, was used as a reference strain (Figure 1).

**FIGURE 1:**
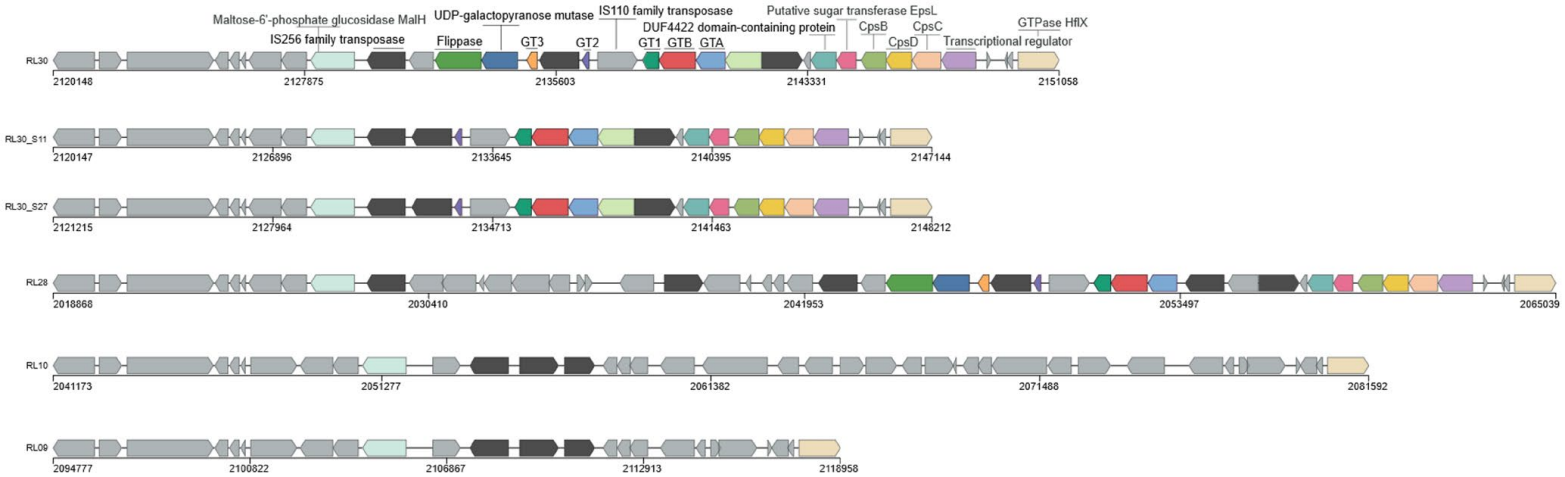
Genomic organisation of the Wzx/Wzy‐dependent cell wall polysaccharide (CWPS) locus across *Lactobacillus crispatus* strains and evolved isolates. Schematic representation of the genomic region encompassing the ~18.7 kb CWPS locus and its neighbouring genes in *L. crispatus* RL09, RL10, RL28, RL30 and two RL30‐derived isolates, S11 and S27. The CWPS locus encodes machinery for a Wzx/Wzy‐dependent glycan biosynthesis, including multiple glycosyltransferases, a flippase, UDP‐galactopyranose mutase, Wzy polymerase and regulatory genes. Within this locus, the putative internal mobile module (~13 kb) flanked by IS256 family transposases and containing an additional internal IS256 element, harbours the GT1‐3 fragments. RL09 and RL10 lack GT1‐3 and CWPS genes (with three IS256 elements in their place), while RL28 has GT1‐3 and additional glycosylation‐related genes, highlighting inter‐strain CWPS diversity. Both evolved strains, obtained through serial propagation, show a partial deletion (~4.1 kb) of the internal mobile module encompassing GT3 and adjacent genes. Genes are coloured according to their functional annotation.

The three GT fragments are located within an approximately 18.7 kb cell wall polysaccharide (CWPS) locus consistent with a Wzx/Wzy‐dependent glycosylation system (Figure 1, Table S1). The locus encodes all essential components of a canonical system, including a priming glycosyltransferase, multiple glycosyltransferases (GT1‐3 and others), flippase, an oligosaccharide polymerase (Wzy), and the regulatory tyrosine kinase module CpsB/CpsC/CpsD (Deo et al. 2019; Rismondo et al. 2021; Butorac et al. 2021). Within the CWPS locus, multiple IS256‐family elements are interspersed, suggesting potential structural plasticity. GT1‐3 are encompassed by a ~13 kb segment flanked by two IS256 transposases, with an additional internal IS256 element, forming a putative mobile internal module. Strains RL09 and RL10 lack GT1‐3 and the entire CWPS locus; three IS256 elements occupy the corresponding genomic region. By contrast, RL28 encodes GT1‐3 in an organization similar to RL30 but with additional glycosylation‐related genes.

### 2.2 Partial Loss of the CWPS Locus During In Vitro Serial Propagation

The ~18.7 kb CWPS locus in *L. crispatus* RL30 contains multiple IS256‐family transposases, suggesting potential structural plasticity. Given the putative role of this locus in host interaction, we hypothesised that it might be unstable under in vitro conditions, where selective pressures from the vaginal environment are absent. To test this, *L. crispatus* RL30, harbouring a complete CWPS locus, was subjected to serial propagation in MRS medium (Figure 2). After 10 propagations, screening by PCR showed that an isolate (RL30_S11) had lost the GT3 fragment. Propagation continued until the 26th passage, which yielded a second isolate without the GT3 fragment (RL30_S27). Whole‐genome sequencing confirmed that both strains had lost a ~4.1 kb segment, encompassing GT3, the flippase, a UDP‐galactopyranose mutase, and an adjacent hypothetical protein (Figure 1). Minor isolate‐specific variations were detected, but none disrupted annotated genes (Table S2). The shared deletion thus represents the only functionally relevant genomic alteration acquired during propagation.

**FIGURE 2:**
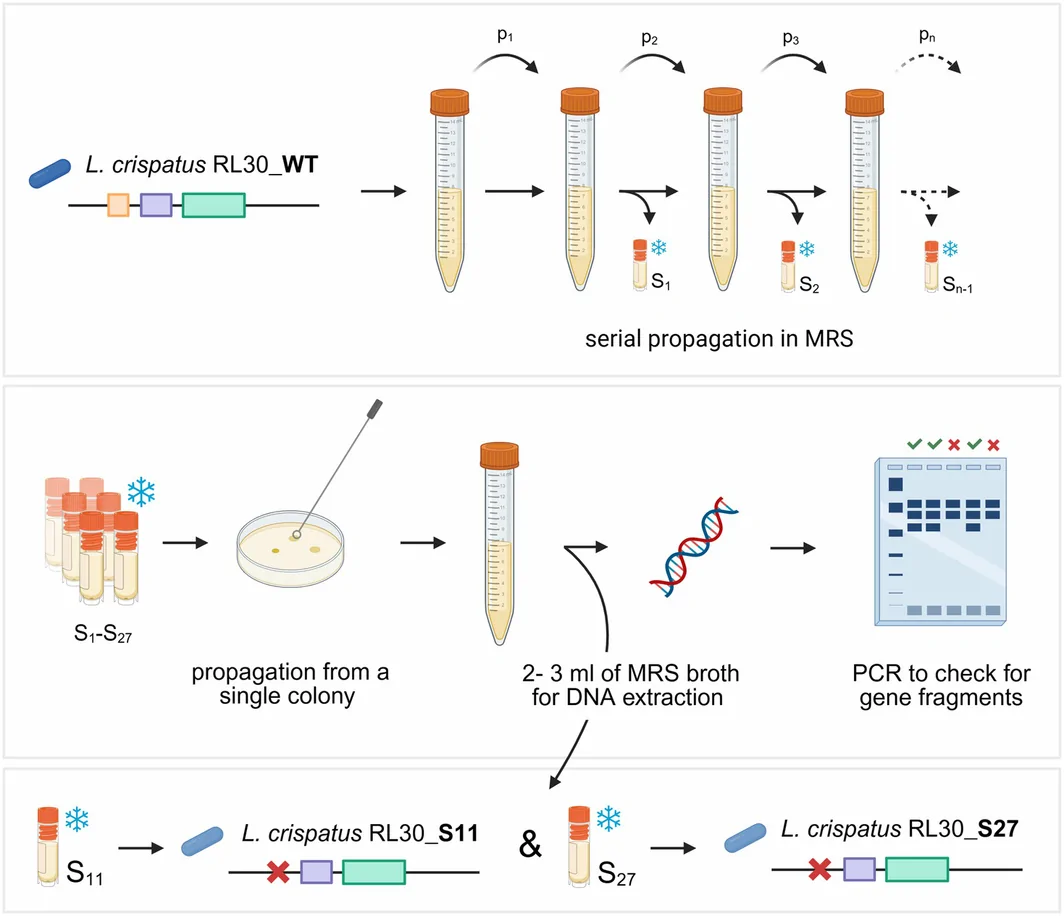
Graphical representation of the serial propagation experiment with *Lactobacillus crispatus* RL30 (WT) harbouring the three glycosyltransferase fragments (GT1‐3). Two evolved strains, RL30_S11 and RL30_S27 were obtained which both lost the GT3 fragment. Yellow, Purple and green boxes indicate GT3, GT2 and GT1 respectively. Created in https://BioRender.com.

### 2.3 Evolved Strains Show a Reduced Thickness of the Peptidoglycan Layer

To assess whether partial CWPS locus loss affected cell wall architecture, RL30 WT and derived isolates S11 and S27 were examined by transmission electron microscopy (TEM). A statistically significant reduction in the peptidoglycan thickness was observed in both evolved strains (Figure 3). The average peptidoglycan thickness in WT was 29.2 ± 6.1 nm, compared to 21.9 ± 5.3 nm and 23.2 ± 4.5 nm for S11 and S27, respectively. The S‐layer showed a trend toward reduced thickness in the evolved isolates; however, this was not statistically significant.

**FIGURE 3:**
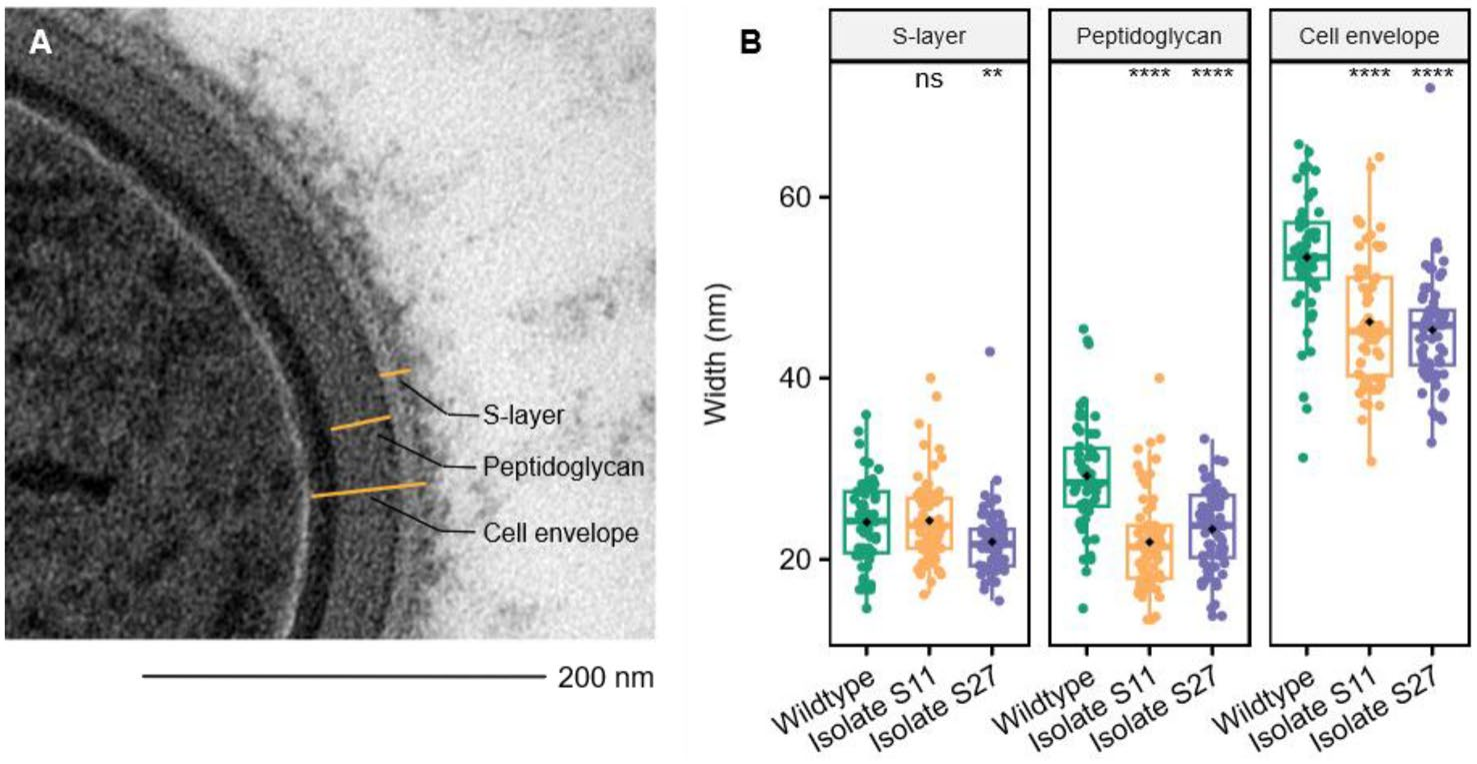
(A) Representative TEM image of a bacterial cell using potassium cyanoferrate staining, indicating the cell envelope, peptidoglycan layer and S‐layer. Red lines indicate the region used for quantitative analysis. (B) Measurements of the different cell wall structures from TEM analysis. Boxplot showing the distribution of peptidoglycan thickness (nm) in *L. crispatus* RL30 wildtype and evolved isolates. Each dot represents a measurement of an individual cell (min. four per cell, min. 14 for each strain). The WT strain has a significantly thicker peptidoglycan layer compared to the two evolved strains (ns: *p* > 0.5, **p ≤ 0.01, ****p ≤ 0.0001; Wilcoxon rank sum test).

CWPS extracts showed no observable differences in the carbohydrate‐associated region by Fourier‐transform infrared spectroscopy (FT‐IR) (Figure S1). Similarly, hydrolysed CWPS extracts showed a comparable qualitative monosaccharide profile by high‐performance anion‐exchange chromatography with pulsed amperometric detection (HPAEC‐PAD), with rhamnose, glucosamine, galactose, glucose, and ribose detected (Figure S1).

### 2.4 Molecular Dynamics Simulations Indicate a Stabilising Role for GT3 in the Glycosyltransferase Complex

The CWPS locus encodes a glycosyltransferase complex split across three gene fragments (GT1‐3). The three fragments align with a single family 2 A‐fold GT gene of the *L. crispatus* ; GT1 and GT2 harbour the core GT fold domain and active site residues (GT1: Tyr12, Asp40, Asp90, Asp92; GT2: Asp200) (Van der Veer et al. 2019, Figure S2). To investigate the structural consequences of GT3 loss, we performed molecular dynamics (MD) simulations comparing GT12 and GT123 complexes. Both configurations formed stable complexes capable of accommodating the common sugar donor UDP within their active domain. The root mean square fluctuation (RMSF) analysis demonstrated that GT12 exhibits higher flexibility in four distinct regions (Figure 4A). Notably, residues 185–195, which are in proximity to catalytic residue Asp200, showed increased flexibility in GT12. The probability distribution of the distance between Asp200 and the UDP‐phosphate tail shows that in GT12 it remains consistently above 0.5 nm, suggesting suboptimal substrate positioning relative to the catalytic residue (Figure 4B).

**FIGURE 4:**
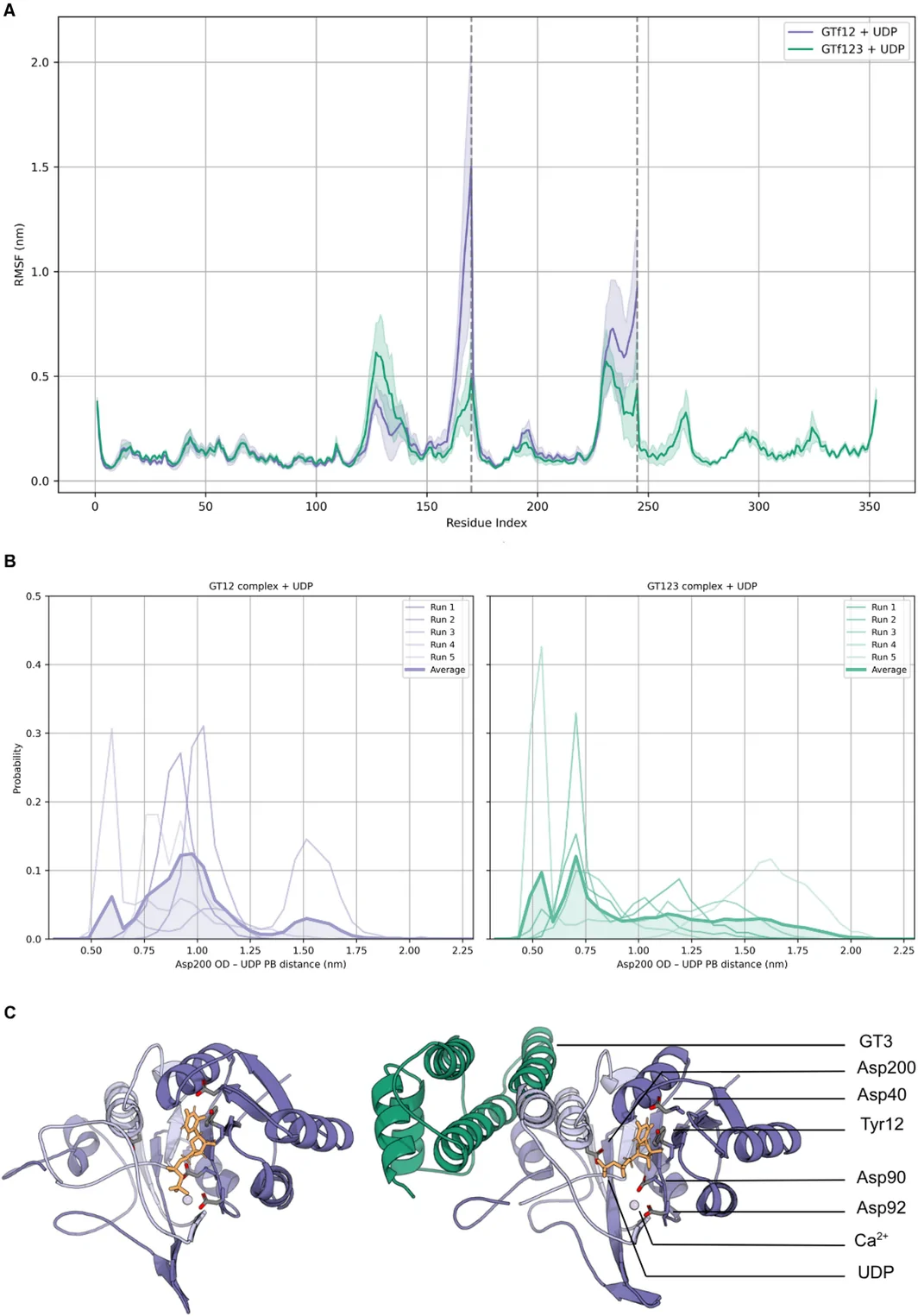
(A) RMSF of the GT12 complex with UDP in the active site (purple) and of the GT123 complex with UDP in the active site (green). The lines display the average Cα RMSF of the systems, with the shaded regions around the lines representing the standard deviation across the replicates. The grey dotted vertical lines indicate the ends of the GT fragments. (B) Probability distribution of the distance between Asp200 and β‐phosphate of UDP. (C) Visual representation of UDP bound in the active site of the GT12 complex on the left and the GT123 complex on the right.

## 3 Discussion

### 3.1 Long‐Read Sequencing Reveals Natural Variation in Cell Wall Glycan Locus

It has been reported that *L. crispatus* strains isolated from *Lactobacillus*‐dominated vaginal microbiota are less likely to harbour glycosyltransferase fragments (GT1‐3), whereas isolates from dysbiotic communities often retained all three fragments (Van der Veer et al. 2019). Our long‐read sequencing revealed that GT1‐3 in *L. crispatus* RL28 and RL30 reside within a ~13 kb internal mobile module flanked by IS256‐family transposases, itself part of a larger ~18.7 kb cell wall polysaccharide (CWPS) locus encoding a canonical Wzx/Wzy‐dependent glycosylation system (Deo et al. 2019; Rismondo et al. 2021; Butorac et al. 2021). The presence of UGM within the locus suggests a role of the CWPS locus in the incorporation of galactofuranose (Gal*f*) residues (Seničar et al. 2020), consistent with a related cluster in *Limosilactobacillus fermentum* D12—sharing the UGM and DUF4422 elements—shown to incorporate Gal*f* in its polysaccharide backbone (Butorac et al. 2021). The enrichment of UGM‐containing cell wall polysaccharide clusters in host‐associated lactic acid bacteria suggests they may contribute to fitness under certain host‐related conditions (Marino and Lederkremer 2014; Deo et al. 2019). Natural variation was evident across strains: RL28 harbored additional glycosylation‐related genes compared with RL30, whereas RL09 and RL10 lacked the full CWPS cluster, retaining only IS256 elements.

### 3.2 Laboratory Evolution Shows Partial Deletion of the CWPS Locus

The abundance of insertion sequences (ISs) in the CWPS locus in *L. crispatus* suggested genomic plasticity (Figure 1), validated in our laboratory evolution experiment. Serial propagation of parental strain RL30 yielded isolates with a ~4.1 kb deletion of the partial internal mobile module encompassing UGM, the flippase, a glycosyltransferase (GT3), and a hypothetical protein. The deletion is flanked by IS256‐family transposases, forming a structure reminiscent of a composite transposon, providing a likely explanation for observed excision. Such transposon‐mediated rearrangements of surface‐associated loci are consistent with earlier studies describing variable and mobile glycosylation gene clusters in lactic acid bacteria (Sybesma et al. 2013; Deo et al. 2019; Butorac et al. 2021); highlighting the evolutionary plasticity of these regions. Notably, no other functionally consequential genomic changes were detected. This strongly indicates that the internal mobile module represents a prominent genetic target for adaptive change in the tested laboratory conditions in the absence of the host or other microbiota.

Why this specific segment is preferentially lost remains unclear. Glycosylation of the outer cell wall is metabolically demanding, requiring sugar activation and membrane translocation (Jayathilake et al. 2017). Loss of part of the CWPS locus could reduce metabolic burden or alter surface properties in a way that offers a selective advantage in the conditions of serial propagation, namely prolonged low‐pH exposure, nutrient depletion, and extended stationary phase.

### 3.3 Partial CWPS Loss Alters the Cell Wall Architecture

TEM imaging showed a reduced peptidoglycan layer in the derived strains, confirming that the deletion has a measurable effect on the cell wall architecture. FT‐IR and HPAEC‐PAD were applied as qualitative screening approaches to assess overall CWPS composition and revealed no gross differences between WT and derived strains. It should be noted that these methods, as applied, do not resolve structural isomers or fine structural differences. The deletion of the UGM is expected to affect the interconversion between galactopyranose (Gal*p*) and galactofuranose (Gal*f*), which cannot be distinguished by the applied HPAEC‐PAD method and was not resolved by FT‐IR. Resolving these configurational consequences will require complementary methods that distinguish Gal*p* from Gal*f*, such as ^1^H/^13^C NMR, permethylation‐GC–MS analysis, or Gal*f*‐specific labelling, which we identify as the next step in characterising the structural impact of the locus (Köplin et al. 1997; Marino and Lederkremer 2014).

Loss of the GT3 fragment, or flippase, may also affect polymerisation efficiency, chain length, and branching of the CWPS, potentially contributing to the altered peptidoglycan thickness observed in TEM. Together the MD simulations indicate that GT3 absence increases active site flexibility and may impair catalytic efficiency. However, the functional impact remains uncertain, especially since Asp200 acts through a single displacement mechanism in which proximity of the phosphate tail may not be strictly required for catalysis.

### 3.4 CWPS Locus Variation in the Context of Vaginal Microbiome Ecology

Our findings support the view that part of the CWPS locus can function as an adaptive module in *L. crispatus*. Although we did not directly assess functional consequences, retention or deletion of the mobile element may influence immune evasion or bacteriophage sensitivity, as alterations in Wzx/Wzy‐dependent loci have been shown to affect both in Lactobacillaceae (Ainsworth et al. 2014; Theodorou et al. 2019). The association of GT1–3 with dysbiotic communities (Van der Veer et al. 2019) and the presence of similar loci in *L. jensenii* linked to preterm birth (Nori et al. 2023) suggest that CWPS configuration influences ecological fitness during changes in the vaginal microbiome. Recent work by Croatti et al. (2026) concerning the same locus CWPS in *L. crispatus* does provide direct functional evidence for its biological relevance (Appendix S1). Deletion of the priming glycosyltransferase *epsE* disrupted extracellular polysaccharide synthesis and altered adhesion capacity and immune modulation. As *epsE* is required for initiation of the repeat‐unit assembly, the perturbation effectively abolishes CWPS production. In contrast, the transposon‐mediated excision in our study resulted in a modified rather than absent CWPS layer, consistent with our observation of altered cell wall architecture without gross compositional changes. Future work should (i) determine the precise CWPS structures and Gal*f* content; (ii) further examine how CWPS variation affects epithelial adhesion, immune activation, and phage susceptibility; and (iii) monitor *locus* dynamics in longitudinal human cohorts.

## 4 Conclusion

In this study, we resolve the genomic context of the GT1‐3 glycosyltransferase gene fragments in *L. crispatus* and demonstrate that they are part of a structurally plastic ~18.7 kb Wzx/Wzy‐dependent cell wall polysaccharide (CWPS) locus. Serial propagation showed that this locus can be rapidly remodelled, with measurable consequences for peptidoglycan thickness. These findings establish a direct link between CWPS locus variation and cell wall architecture in *L. crispatus* , and provide a basis for further investigation into the role of strain‐level glycosylation diversity in the vaginal microbiome.

## 5 Experimental Procedures

### 5.1 Bacterial Strains and Culture Conditions

The *L. crispatus* strains used in this study are listed in Table 1. All cultures were grown anaerobically at 37°C in MRS broth or on MRS agar. Full culture conditions, media composition, and anaerobic handling procedures are described in Methods S1.

**TABLE 1:** List of *Lactobacillus crispatus* strains used in this study for either wet‐lab experiments and/or genomic sequencing and comparative analysis of the CWPS locus. The strains, except for the strains produced in this study, have been deposited at the Westerdijk Institute, Utrecht, The Netherlands.

Species	Strain	Source	NCBB database ID	References
*L. crispatus*	RL07	Human vagina	NCCB 100713	Dols et al. (2016), Van der Veer et al. (2019)
*L. crispatus*	RL09	Human vagina	NCCB 100714	Dols et al. (2016), Van der Veer et al. (2019)
*L. crispatus*	RL10	Human vagina	NCCB 100715	Dols et al. (2016), Van der Veer et al. (2019)
*L. crispatus*	RL28	Human vagina	NCCB 100728	Dols et al. (2016), Van der Veer et al. (2019)
*L. crispatus*	RL30 (WT)	Human vagina	NCCB 100730	Dols et al. (2016), Van der Veer et al. (2019)
*L. crispatus*	RL30 variant 1 (S11)	Human vagina	—	This study
*L. crispatus*	RL30 variant 2 (S27)	Human vagina	—	This study

### 5.2 Serial Propagation

*L. crispatus* RL30 was serially passaged 26 times in MRS broth with 1:100 (v/v) dilutions every 48–64 h. Growth was monitored by OD_600_ and pH at each passage. Additionally, at each passage, cultures were cryopreserved at −70°C. Loss of the GT gene fragments was screened by multiplex PCR after DNA extraction. Full passaging and PCR protocols are described in Methods S2 and S4.

### 5.3 DNA Isolation

Genomic DNA was extracted from 2 to 3 mL of liquid culture using the GenElute Bacterial Genomic DNA kit, following the manufacturer's protocol for Gram‐positive bacteria. DNA purity and concentration were assessed by spectrophotometry. Full extraction details are provided in Methods S3.

### 5.4 Long‐Read Sequencing and Genomics Analysis

Genomic DNA from strains RL09, RL10, RL28, RL30, and the derived variants was sequenced using Oxford Nanopore Technology (GridION, R10.4.1 flow cell, Kit 14). For RL30, a hybrid assembly combining previously generated Illumina short reads and new long reads was used. Functional annotation and visualisations were made from the generated genome assemblies. Full bioinformatic parameters and software versions are provided in Methods S5. Raw reads and assemblies are deposited in ENA under accession PRJEB106179.

### 5.5 Transmission Electron Microscopy

*L. crispatus* cultures were harvested after 24 h growth and processed for TEM at the Wageningen Electron Microscopy Centre using standard potassium cyanoferrate staining. Cell wall layer thickness was quantified from TEM images in ImageJ Schneider et al. (2012); full image analysis measurement procedures can be found in Methods S6. Four measurements per image were taken across 14–17 images per strain. Statistical comparisons were performed using the Wilcoxon rank‐sum test.

### 5.6 Cell Wall Polysaccharide Extraction and Analysis

CWPS were extracted from 1 L batch cultures of RL30 wildtype and derived variants following the protocol from Sadovskaya and Guérardel (2019) with slight alterations. CWPS extracts were analysed by FT‐IR spectroscopy and additionally hydrolysed and measured by HPAEC‐PAD to determine the monosaccharide composition. This was done in two biological replicates. Additional details can be found in Methods S7–S9.

### 5.7 Molecular Dynamics Simulations

GT complex structures (GT12 and GT123) were predicted using AlphaFold3 Abramson et al. (2024), with UDP docked into predicted binding pockets identified with Fpocket Le Guilloux et al. (2009) and SMINA Koes et al. (2013). Both systems were simulated for 5 μs using GROMACS2023.3 Abraham et al. (2015, 2023) with the CHARMM36m Huang et al. (2017) and TIP3P water model Jorgensen et al. (1983). RMSD and per‐residue RMSF were computed from Cα atoms. Full simulation setup, equilibration protocol, and analysis scripts are provided in the Methods S10.

## Author Contributions

**P. Achterberg:** conceptualization, writing – original draft, investigation. **J. Vroom:** investigation. **R. Kort:** conceptualization, funding acquisition, writing – review and editing, supervision, methodology. **M. Lekkerkerker:** investigation, writing – review and editing, methodology. **S. Illidge:** investigation. **M. C. M. van Loosdrecht:** writing – review and editing, supervision, funding acquisition. **R. Y. Hertzberger:** supervision, writing – review and editing. **J. Vreede:** writing – review and editing, supervision. **L. Koelemeijer:** investigation.

## Funding

This work was supported by Nederlandse Organisatie voor Wetenschappelijk Onderzoek, Spinoza Prize, Vrije Universiteit Amsterdam.

## Disclosure

Copyright and Permissions Statement: No copyright permissions were required for any figures, tables, or other material reproduced or adapted in this manuscript.

## Ethics Statement

The authors have nothing to report.

## Conflicts of Interest

The authors declare no conflicts of interest, except that R. Kort is listed as inventor on patent application P36697NL00 (“Mixture of *Lactobacillus crispatus* strains”) relating to the subject matter of this work.

## Supporting information


**Table S1:** Proteins encoded in the *Lactobacillus crispatus* RL30 cell wall polysaccharide locus.
**Table S2:** Genomic differences between L. crispatus RL30 wildtype and evolved isolates S11 and S27 outside the cell wall polysaccharide locus.
**Figure S1:** Structural properties of the cell wall polysaccharides (CWPS) of Lactobacillus crispatus RL30 wildtype and evolved strains S11 and S27. Left: FT‐IR spectrum of extracted CWPS, shown in replicates (1) and (2). The grey area (950–1200 cm−1) indicates the region for carbohydrates. Right: HPAEC‐PAD spectra the hydrolysed CWPS.
**Figure S2:** A. Multiple sequence alignment of the three GT fragments against the GTA observed in *Lactobacillus crispatus* 125‐2‐CHN reference strain.
**Methods S1‐S10:** Supplementary experimantal procedures, including Table S3.
**Appendix S1:** BLAST analysis of GT1‐3 and CWPS locus genes across specific *Lactobacillus* *crispatus* strains, including Table S4.

## Data Availability

Raw long‐read sequencing data and genome assemblies generated in this study have been deposited in the European Nucleotide Archive (ENA) at EMBL‐EBI under project accession PRJEB106179 (https://www.ebi.ac.uk/ena/browser/view/PRJEB106179). Input parameters, starting structures, and topology files for the molecular dynamics simulations are available upon request.
