# Supplementary material for: A Mobile Glycosylation Locus Modulates Cell Wall Architecture in Lactobacillus crispatus
Source: Mol Microbiol. 2026 Sep 10;126(5):473–81. doi: 10.1111/mmi.70112 (PMC13725318; doi:10.1111/mmi.70112)
Supplement: Supplementary file 1 [file MMI-126-473-s001.pdf]

Supporting Information

A mobile glycosylation locus modulates cell wall architecture in *Lactobacillus crispatus*

P. Achterberg, M. Lekkerkerker, L. Koelemeijer, S. Illidge, J. Vroom, R. Y. Hertzberger, J. Vreede, M. C. M. van Loosdrecht, R. Kort*

*Corresponding author: R. Kort (r.kort@vu.nl), Amsterdam Institute for Life and Environment (A-LIFE), Vrije Universiteit Amsterdam, Amsterdam, the Netherlands.

Contents

Table S1. Proteins encoded in the *Lactobacillus crispatus* RL30 cell wall polysaccharide locus.

Table S2. Genomic differences between *L. crispatus* RL30 wildtype and evolved isolates S11 and S27 outside the cell wall polysaccharide locus.

Figure S1. Structural properties of the cell wall polysaccharides (CWPS) of *L. crispatus* RL30 wildtype and evolved isolates S11 and S27.

Figure S2. Multiple sequence alignment of the three GT fragments against the GTA of the *L. crispatus* 125-2-CHN reference genome.

Methods S1–S10. Supplementary experimental procedures, including **Table S3** (oligonucleotides used for multiplex PCR).

Appendix S1. BLAST analysis of GT1–3 and CWPS locus genes in selected *L. crispatus* strains, including **Table S4** and the query sequences used.

References cited in the Supporting Information.

Supplementary Tables

Table S1. Proteins encoded in the *Lactobacillus crispatus* RL30 cell wall polysaccharide (CWPS) locus. Entries are listed in their order in the locus.

RefSeq ID / UniParc ID	Predicted product of encoded protein
WP_005727505.1 / UPI0001EC2968	IS256 family transposase
WP_005730042.1 / UPI000280BB19	Hypothetical protein
WP_005727389.1 / UPI0001EC296D	Polysaccharide biosynthesis protein C-terminal domain-containing protein / flippase (*wzx*)
WP_005723855.1 / UPI0001B29BCA	UDP-galactopyranose mutase

RefSeq ID / UniParc ID	Predicted product of encoded protein
– / UPI000280BB18	Glycosyltransferase group 2 family protein / GT3
– / UniRef90_P35880	IS256 family transposase
– / –	– / GT2
WP_005722630.1 / UPI0001B2A024	IS110 family transposase
WP_005727395.1 / UPI0001EC2972	Glycosyltransferase 2-like domain-containing protein / GT1
WP_005723852.1 / UPI0001B29BC7	Glycosyl transferase family 1 domain-containing protein / GTB
WP_005723850.1 / UPI0001B29BC6	Glycosyltransferase 2-like domain-containing protein / GTA
– / UniRef90_K1N8B8	Oligosaccharide repeat unit polymerase (*wzy*)
– / UniRef90_P35880	IS256 family transposase
– / UPI000280B3A8	Hypothetical protein
WP_005723848.1 / UPI0001B29BC4	DUF4422 domain-containing protein
WP_005727402.1 / UPI0001EC2977	Putative sugar transferase *epsL**
WP_005727403.1 / UPI0001EC2978	Tyrosine-protein phosphatase (*cpsB*)
WP_005723845.1 / UPI0001B29BC1	Tyrosine-protein kinase (*cpsD*)
WP_005723844.1 / UPI0001B29BC0	Capsular polysaccharide biosynthesis protein (*cpsC*)
WP_005727404.1 / UPI0001EC2979	Transcriptional regulator

* >70% identity with the priming glycosyltransferase (*epsE*) of *L. gasseri* DSM 14869 (gene accession N506_0400).

Table S2. Genomic differences between *L. crispatus* RL30 wildtype and evolved isolates S11 and S27 outside the cell wall polysaccharide locus.

Genomic feature	Wildtype RL30	Isolate 1 (S11)	Isolate 2 (S27)
YSIRK-type signal peptide protein	Full length	Full length	Shorter variant (720 bp)
IS1182 family transposase	Absent	Absent	Present (1713 bp) at 1573798–1575510 bp

Genomic feature	Wildtype RL30	Isolate 1 (S11)	Isolate 2 (S27)
IS256 family transposase	Absent	Present (1179 bp) at 2219905–2221083 bp	Present (1179 bp) at 2220973–2222151 bp

Supplementary Figures

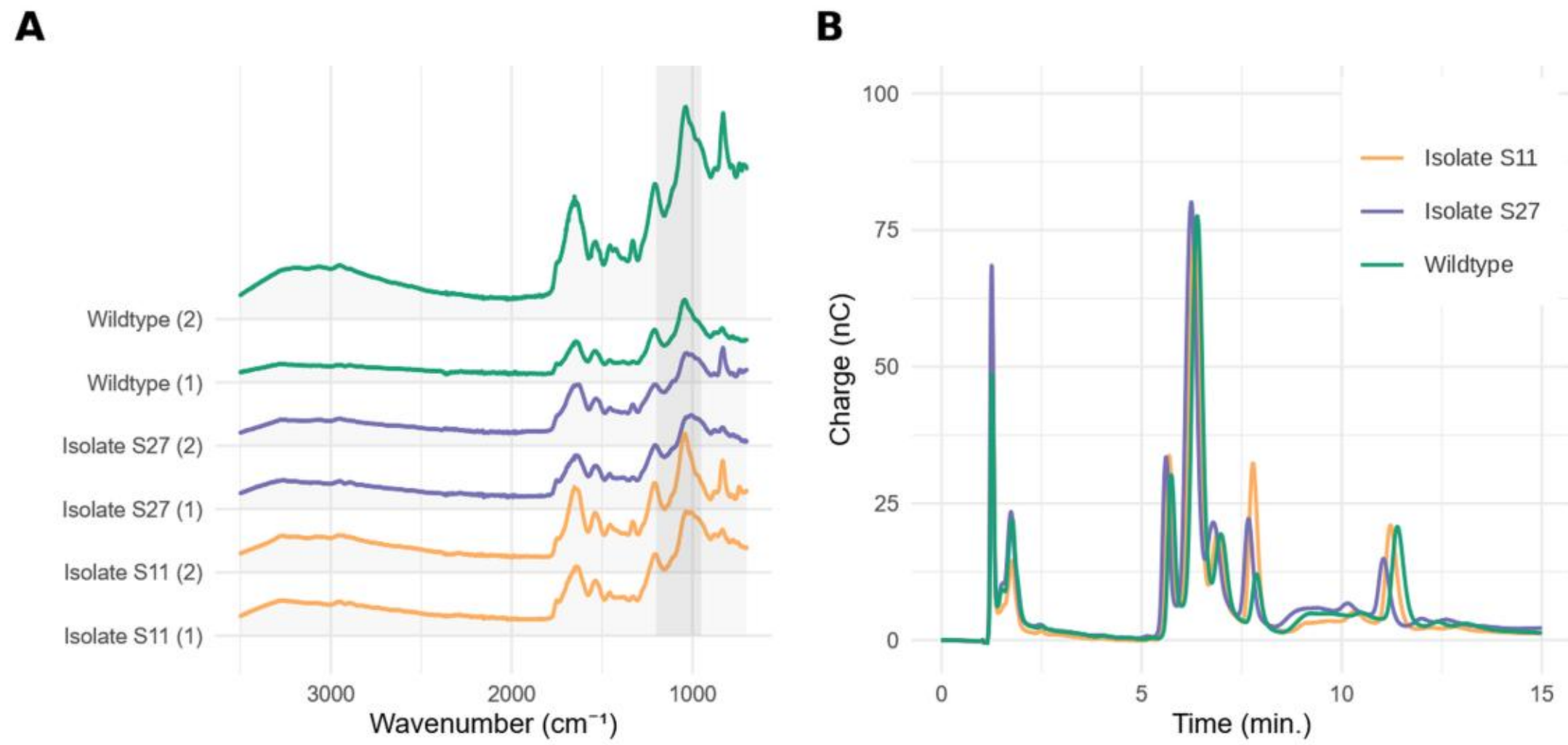

Figure S1. Structural properties of the cell wall polysaccharides (CWPS) of *Lactobacillus crispatus* RL30 wildtype and evolved isolates S11 and S27. (A) FT-IR spectra of the extracted CWPS, shown for two biological replicates (1) and (2) per strain. The grey band (950–1200 cm^{-1}) indicates the carbohydrate region. (B) HPAEC-PAD chromatograms of the hydrolysed CWPS.

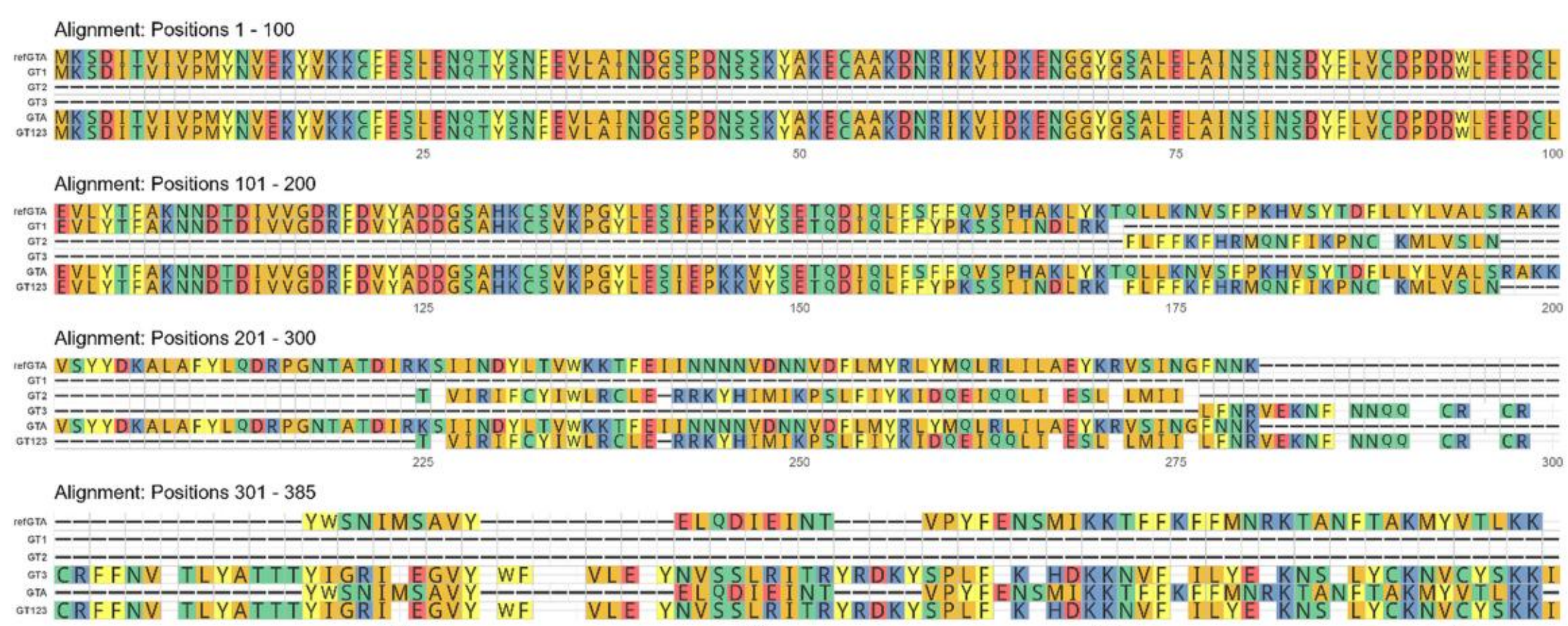

Figure S2. Multiple sequence alignment of the three GT fragments (GT1, GT2 and GT3) against the GTA of the *Lactobacillus crispatus* 125-2-CHN reference genome. refGTA, reference GTA sequence; GT123, the concatenated fragments.

Supplementary Methods

Methods S1. Bacterial strains and (initial) culture conditions

All bacterial growth experiments were performed anaerobically at 37°C. Anaerobic conditions were maintained in anaerobic jars (Oxoid modified 3, HP0011a or an airtight metal jar) with anaerobic gas-generating sachets (AnaeroGen 2.5 or 3.5L; ThermoScientific, Waltham, MA, USA) added or flushed three times with gas (95% N_2; 5% CO_2) after creating a vacuum, before sealing. Handling of the bacterial cultures, including inoculation and transfers, was performed under sterile conditions using standard aseptic techniques under ambient atmospheric conditions. Immediately after handling, cultures were transferred to anaerobic jars to minimise oxygen exposure. Throughout all experiments and each procedural step, negative controls were included and inspected for microbial contamination.

Bacterial cultures were propagated from -70°C NYCIII (New York City Broth III) or MRS (De Man Rogosa Sharpe) glycerol stocks (~20% w/w) on MRS agar (1.5%) plates for at least 48h. Pre-cultures of 2 mL MRS were inoculated from the plate by streaking several colonies and incubated for 22-26h in screw-capped tubes with loose lids to allow gas transfer between the tubes and the jar. The inoculation plates and pre-cultures were always freshly prepared before each growth experiment.

For the liquid media and agar plates, MRS broth (GranuCult® plus, Merck KGaA, Darmstadt, Germany) was used or MRS broth from Carl Roth (Karlsruhe, Germany) with the addition of 1 ml/L Tween80 (Sigma-Aldrich, Merck KGaA). Different from what was specified by the manufacturer's instructions, it was sterilised for 20 minutes at 110°C. For agar plates, 15 g/L agar was added (Difco™ Agar, BD, Maryland, USA). Plates and liquid media were stored at 4°C until usage.

Methods S2. Serial propagation of *L. crispatus* RL30

For serial propagation, *L. crispatus* RL30 was recovered from cryopreserved stock as described previously and directly subjected to serial passaging in MRS broth (Figure 2, main text). Each passage was initiated every 48-64h by a 1:100 (v/v) dilution of the previous culture into 3 mL prewarmed (37 °C) MRS broth in screw-capped tubes. Optical density at 600 nm (Novaspec III spectrophotometer, Amersham Biosciences, Cambridge, UK) and pH (HI2210 pH meter, Hanna Instruments, Woonsocket, USA) were measured before and after each passage to validate proper growth of the bacteria. At each passage, 1 mL of culture was mixed with 0.5 ml 60% (v/v) glycerol for cryopreservation at -70 °C. In total, twenty-six serial passages were performed. From each glycerol stock, plates were inoculated from which one single colony was propagated in liquid media to make genotypic uniform glycerol stocks and to screen for the loss of the GT fragments by multiplex PCR after DNA extraction.

Methods S3. DNA isolation of cultures

For the DNA extraction, 2 to 3 ml of liquid microbial culture was harvested by centrifugation at ≥ 12000 x g (Sigma 1-14K, Sigma Laborzentrifugen GmbH, Osterode am

Harz, Germany) for 2 min., after 24h of incubation in MRS as described in the section "Bacterial strains and (initial) culture conditions", albeit without pre-culture and using a single colony. Genomic DNA was extracted using the GenElute Bacterial Genomic DNA kit (Sigma-Aldrich, Cat. No. NA2110) according to the manufacturer's protocol for Gram-positive bacteria. Lysozyme at 45 mg/mL (Sigma-Aldrich, Cat. No. L4919) is not provided in the kit. The purity and concentration of the extracted DNA were measured with NanoDrop® ND-1000 Spectrophotometer at 260nm (ThermoFisher Scientific) and were between 61.5 and 151.3 ng/µL.

Methods S4. Multiplex PCR

Previously designed oligonucleotide primers (Van der Veer *et al.*, 2019) for three specific gene fragments were used for the multiplex PCR (Table S3). The primers were resuspended in 1x TE buffer (10 mM Tris-HCl, 1 mM EDTA, pH 7.0) to a working concentration of 100 µM. PCR reactions were prepared in a total volume of 50 µL using the DreamTaq™ Hot Start PCR MasterMix (ThermoFisher Scientific, Waltham, USA) according to the manufacturer's instructions. The reaction mixture included 25 µL DreamTaq™ Hot Start Green PCR MasterMix, 18 µL primermix and 1 µL of bacterial DNA, extracted as described in a previous section. The optimised primermix consisted of 2.5 µM, 1.25 µM, 1.87 µM of forward and reverse primer of fragment 1, 2 and 3, respectively.

PCR amplifications were executed in a Westburg Biometra T-advanced PCR machine (Analytik Jena, Jena, Germany) using the following cycling conditions: initial denaturation step at 95 °C for 3 minutes, followed by 30 cycles of denaturation at 95 °C for 30 seconds, annealing at 51 °C for 30 seconds and extension at 72 °C for 30 seconds. A final extension was performed at 72 °C for 5 minutes. The PCR products were run on a 30 mL, 2% agarose gel (CSL-AG500 LE Multi-Purpose Agarose, Cleaver Scientific, Rugby, UK) with 4 µL Roti®GelStain (Roth, Karlsruhe, Germany), and 1x Tris-Acetate-EDTA (TAE) buffer. The electrophoresis was performed at 110V for 25-30 minutes, and bands were visualised with an UV analyser (Transilluminator Model M-20, Appligene, USA). Negative controls without bacterial DNA, positive controls containing bacterial DNA of *L. crispatus* RL30 wildtype and DNA ladder (100 bp DNA ladder, New England BioLabs, Ipswich, MA, USA) were included in each run.

Table S3. Oligonucleotides designed for the multiplex PCR of the three GT gene fragments (Van der Veer *et al.*, 2019).

Primer name	Oligonucleotide sequence (5'→3')	T_m (°C)	GC-content	Fragment length
Fragment 1 (fw)	AGACATAACCGTAATTGTTCCAA	55.3	35%	360 bp
Fragment 1 (rv)	TACACATCAAATCGATCCCC	55.3	45%	360 bp
Fragment 2 (fw)	TTTTTCAAGTTTCACCGCAT	51.2	35%	150 bp

Primer name	Oligonucleotide sequence (5’→3’)	T_m (°C)	GC-content	Fragment length
Fragment 2 (rv)	GCGAGGGCTTTATCATAATATG	56.5	41%	150 bp
Fragment 3 (fw)	TGTCGATAATAATGTCGATAATAATGT	55.9	26%	250 bp
Fragment 3 (rv)	GCAGTAAAGTTAGCTGTTTTTCTATTC	58.9	33%	250 bp

Methods S5. Long-read sequencing and genomic analysis of *L. crispatus* strains

Extracted DNA from *L. crispatus* strains RL09, RL10, RL28, RL30, and variants obtained in this study (Table 1, main text) was sent to Hologenomix B.V. (Delft, The Netherlands) for sequencing and genome assembly. For the reference strain *L. crispatus* RL30, a hybrid approach was employed, combining short-read sequencing (from previous work using Illumina MiSeq) and newly generated long reads to improve assembly accuracy. For the other strains, solely long-read sequencing was utilised to generate complete genome assemblies. Both the raw long-reads and genome assemblies have been deposited in the European Nucleotide Archive (ENA) at EMBL-EBI under the project accession PRJEB106179 (https://www.ebi.ac.uk/ena/browser/view/PRJEB106179).

The strains were sequenced using long-read Oxford Nanopore technology, following the manufacturer’s protocol for microbial isolates, using the Rapid Barcoding Kit from the same company in its multiplex format with 96 different barcodes, with the chemistry Kit 14 (SQK-RBK114.96). After preparation, the samples were sequenced in a Flow Cell R10.4.1 (FLO-MIN114) in the sequencer GridlON for a total of 12 hours. Basecalling was performed with MinKNOW (v.5.9.12) and reads with $Q < 10$ were discarded. Read quality was assessed with NanoPlot (v.1.43.0). Assemblies were generated using three independent assemblers Flye v.2.9.5-b1801 (Kolmogorov *et al.*, 2019), Raven v. 1.8.3 (Vaser and Šikić, 2021), Miniasm+ Minipolish v.0.3-r179 (Li, 2016) and reconciled with Trycycler (Wick *et al.*, 2021) to obtain high-quality consensus assemblies. Polishing after reconciliation was done with Medaka v.2.0.1 (Oxford Nanopore Technologies, 2018) for long reads, and Polypolish v.0.6.0 (Wick and Holt, 2022; Bouras *et al.*, 2024) for short reads, to further improve base-level accuracy. Manual curation and visualisation were conducted using Bandage (Wick *et al.*, 2015), assemblies were included based on two main criteria: the presence of a single large contig representing the chromosome, the presence of a smaller contig for the plasmid (when applicable). Assembly completeness and contamination were assessed using CheckM v.1.2.2 (lineage_wf workflow) (Parks *et al.*, 2015). Functional annotation of the consensus assemblies was performed using Bakta v.1.9.4, which also generated GFF3 files used in the visualisations (Schwengers *et al.*, 2021).

Genome alignments and visualisations were generated with Lovis4u (Egorov and Atkinson, 2025). The tool was installed and executed via Pixi (v0.63.2) using default dependencies (Arts *et al.*, 2026). A custom locus annotation table and feature annotation file were used to respectively define the region of interest and highlight genes of interest.

Methods S6. Transmission electron microscopy and ImageJ analysis

L. crispatus bacterial cultures were inoculated in 3 ml of MRS directly from the glycerol stock, harvested after a 24h incubation and transferred (at 4 °C) the same day for sample preparation and transmission electron microscopy (TEM) imaging, performed at the Wageningen Electron Microscopy Centre (WEMC), following their standard protocol for potassium cyanoferrate electron microscopy. All samples were processed and imaged within the same experimental batch to minimise technical variation.

TEM images were analysed using ImageJ (version 1.54f, Java 1.8.0_322) (Schneider *et al.*, 2012). Before analysis, the image scale was calibrated by referencing the embedded scale bar in each image. Lines were drawn from the cell membrane through the S-layer to the outer cell wall boundary. Intensity profiles (gray value vs. distance) were generated with the function 'Plot Profile'. Local minima in the profile were used to determine the location of the cell membrane and S-layer, while the plateau region at the end, corresponding to the background intensity, marked the outer cell wall boundary. Each TEM image contributed four evenly distributed measurements. Resulting dataset sizes were: RL07 (56 measurements from 14 images), RL09 (60 from 15 images), RL28 (60 from 15 images), RL30-WT (56 from 14 images), RL30_S11 (64 from 16 images), and RL30_S27 (68 from 17 images).

Quantitative data were compiled in Excel, and for each bacterial strain, the distance from the cell membrane and S-layer to the outer cell wall was determined, referred to as the peptidoglycan and S-layer, respectively. Normal distribution of the samples was checked with the Shapiro-Wilk test, and as most of the samples were non-normally distributed a Wilcoxon rank-sum test was performed to analyse differences between the strains. Statistical analysis and generation of plots were performed in R version 4.4.0 (2023-04-24 ucrt) (R Core Team, 2021) with the following packages: readxl (v1.4.5) (Wickham *et al.*, 2025a), ggplot2 (v4.0.1) (Wickham *et al.*, 2025b), tidyverse (v2.0.0) (Wickham and RStudio, 2023), tidyr (v1.3.1) (Wickham *et al.*, 2024), and ggpubr (v0.6.2) (Kassambara, 2025). To assess measurement reproducibility, two independent investigators repeated the line-drawing analysis on the same image set and obtained concordant results; the data set reported is from a single investigator.

Methods S7. Extraction of cell wall polysaccharides (CWPS)

Ten millilitres of pure pre-culture of the *L. crispatus* RL30 WT and variant strains (Table 1, main text) were inoculated in 1 L MRS broth and incubated overnight at 37 °C and 50 rpm (Certomat®BS-1, Sartorius Stedim Biotech GmbH, Göttingen, Germany). Cultures were harvested at an optical density (600 nm) of 1.1-1.5 AU and pH of 4.6-4.9, by centrifugation for 10 minutes at 3900 rpm and 4°C (Eppendorf Centrifuge 5810R, Eppendorf SE, Hamburg, Germany). Pellets were washed twice by resuspension in 10 mL phosphate-buffered saline (PBS, Sigma-Aldrich) followed by centrifugation under the same conditions as previously described. Wet biomass yield ranged from 1.9 to 3.1 g per culture and was stored at -20°C until the cell wall polysaccharides (CWPS) extraction.

Crude CWPS were extracted following the protocol as developed by Sadovskaya and Guérardel, (2019) for gram-positive bacteria through section 3.1 'Extraction of CWPS) step

2 to step 11 of the same section (Sadovskaya and Guérardel, 2019). An additional step was added before the final freeze-drying (step 11). The obtained dialysate was transferred to a Falcon tube covered with parafilm pierced with small holes and frozen at -80 °C until solid. For the dialysis, SnakeSkin™ Dialysis Tubing 35 mm, 3.5k MWCO (ThermoScientific) was used. The final product (between 7.3 - 22.6 mg) was stored at room temperature until further analysis. The complete process of growing biomass and the cell wall extraction was done in duplicate.

Methods S8. Functional group analysis by FT-IR

To identify differences in the chemical profile of the CWPS extracts of the *L. crispatus* RL30 WT and variants, the Fourier-transform infrared (FT-IR) spectrum was recorded using an FT-IR spectrophotometer (Perkin Elmer, Shelton, USA) in attenuated reflectance (ATR) mode at room temperature with a wavenumber range from 550 cm^{-1} to 4000 cm^{-1}.

Methods S9. Sugar monomer detection by HPAEC-PAD

To analyse the sugar monomers, one set of CWPS extracts were hydrolysed in 1 mL of 1 M HCl in an oven (105 °C) for 8 h. After centrifugation, the supernatant was first filtered through 0.22 µm PVDF filters. The filtrate was evaporated to get rid of water as well as HCl by using nitrogen blowing devices with constant gas flow rate. Water bathing below 50 °C was applied if necessary to speed up the drying process. Eventually, these dry sticky sugar powders were dissolved with 200 µL MilliQ water for sugar monomers injection. High-performance anion-exchange chromatography with pulsed amperometric detection (HPAEC-PAD, a Dionex ICS-5000+, a CarboPac PA20 column, and an AminoTrap precolumn) was applied to analyse the sugar monomers. The standard compounds were run with the samples and contained seven neutral sugars (fucose, rhamnose, galactose, glucose, xylose, mannose, and ribose), two amino sugars (galactosamine and glucosamine), and two uronic acids (galacturonic and glucuronic acid). Automatic integration by the software (Chromeleon 7, ThermoScientific), was manually checked and if needed adjusted.

Methods S10. Molecular dynamics simulations of the glycosyltransferase complex

The initial coordinates of GT12 and GT123 were predicted with AlphaFold 3 (Abramson *et al.*, 2024), based on the amino acid sequence of GT123 as a single chain and a Mn^{2+} ion. Note that we used the divalent ion Ca^{2+} in the MD simulations. From this prediction, the GT12 and the GT123 systems were constructed by labelling the atoms of GT1, GT2 and GT3 as separate chains, and for GT12, removing the atoms of GT3 from the structural model.

To add UDP to the enzyme complexes, we first identified the conserved active site residues via a multiple sequence alignment of various GTs. Then, we searched for potential binding pockets in the structural models produced by alphafold, in the vicinity of the conserved active site residues. To complete the addition of UDP to the GT complexes, we performed molecular docking of UDP within a region in the complex that contained the potential active site. To confirm that the residues in contact with UDP are indeed part of the active site, we checked whether these residues are conserved in the multiple sequence alignment and whether they match with the crystal structure of another GT, (pdb_00001qg8; Charnock and Davies, 1999; Tarbouriech *et al.*, 2001).

The multiple sequence alignment was produced with MAFFT version 7 (Kuraku *et al.*, 2013; Katoh *et al.*, 2019) using the default settings, using different GTs acquired from NCBI (National Center for Biotechnology Information). Only sequences of the GT family-2 proteins were used. To improve the clarity of the alignment visualization, GT family-2 sequences of similar amino acid length were selected to minimise gaps and ensure readability.

Putative ligand-binding pockets were identified using Fpocket through the BioBB version 5.0.0 (BioExcel Building Blocks, Le Guilloux *et al.*, 2009), using a minimum alpha-sphere radius of 3 Å, a maximum radius of 6 Å, and a requirement of at least 35 alpha spheres per pocket. Following the initial detection phase, the pockets were filtered to retain only those cavities with volumes ranging from 500 $Å^3$ to 5000 $Å^3$. This step effectively excluded cavities that were either excessively small or considerably large, as they are unlikely to accommodate the ligands of interest. Next, the pockets were visualised in NGLview version 3.1.4 (Nguyen *et al.*, 2018), and the pocket located within the active domain was extracted. The coordinates of the extracted pocket were then used to define the dimensions of the docking box. The ligands were docked into the defined docking box with SMINA version 1.1.2 (Koes *et al.*, 2013), using an exhaustiveness of 32, an energy range of 5 kcal/mol, and the AutoDock4 scoring function (Huey *et al.*, 2007; Wang *et al.*, 2011). Finally, the ligand pose closest to the relevant active site residues was extracted and used for molecular dynamics simulations.

To prepare the enzyme complexes GT12-UDP and GT123-UDP for molecular dynamics simulations, the complexes were placed in a dodecahedral periodic box with a minimum distance of 1.2 nm between the protein and the box edge. Then, solvent was added, and to maintain charge-neutrality, Na^+ and Cl^- ions were added at a concentration of 0.1 M. All interactions, including UDP, are described by the CHARMM36 force field (Huang *et al.*, 2017) in combination with the TIP3P water model (Jorgensen *et al.*, 1983; Boonstra *et al.*, 2016). Electrostatic interactions were handled by the Particle Mesh Ewald (PME; Darden *et al.*, 1993) method with a 1.2 nm real-space cut-off. Van der Waals interactions were handled with a force-switch modifier between 1.0 and 1.2 nm. A Verlet cut-off scheme with a 1.2 nm neighbour list cut-off was used for neighbour searching (Páll and Hess, 2013).

First, the energy of the system was minimised to a maximum force threshold of 150 kJ/mol nm using the conjugate gradient method, followed by an equilibration of 10 ps molecular dynamics at constant pressure and temperature, with harmonic position restraints with a force constant of 1000 kJ/mol nm^2 in each direction for the non-hydrogen and non-solvent (i.e. water and ions) atoms. Then, 5 microsecond-long production runs were performed at constant pressure of 1 bar and temperature of 310 K and with a time step of 2 fs using LINCS to constrain bonds (Hess *et al.*, 1997). Each run started with random velocities drawn from the Maxwell-Boltzmann distribution at 310 K. Pressure was kept constant with the Parrinello-Rahman barostat at 1 bar (Parrinello and Rahman, 1981; Nosé and Klein, 1983) and temperature was kept constant at 310 K with the velocity-rescaling thermostat (V-rescale; (Bussi *et al.*, 2007).

All simulations were performed using GROMACS version 2023.3 (Abraham *et al.*, 2015; Abraham *et al.*, 2023). Input parameters, starting structures and topology files are available upon request.

Coordinates of the enzyme complex were saved every 5 ps for further analysis, after removal of periodic boundary artefacts. Analysis consisted of computing the root mean square deviation (RMSD) as a function of time and the root mean square fluctuations (RMSF) per residue. The RMSD and RMSF calculations included the carbon alpha atoms, and for the RMSD the 100th frame of each simulation was used as a reference. The RMSF was computed after fitting all frames on the included atoms.

In addition, the interfaces between GT1, GT2 and GT3, and the interaction between UDP and the enzyme complex were inspected visually and computed. Analysis was performed in python scripts, using the MDTraj library (McGibbon *et al.*, 2015). The structural snapshots were generated with PyMOL (The PyMOL Molecular Graphics System, Version 3.0, Schrödinger, LLC).

Appendix S1. BLAST analysis of GT1–3 and CWPS locus genes in selected *L. crispatus* strains

BLAST searches were performed to assess the presence of glycosyltransferase genes GT1, GT2, and GT3, along with additional cell-wall polysaccharide (CWPS) locus genes, across *L. crispatus* strain CTV-05 (LACTIN-V; GCA_000165885.1) and strains BC1, BC3 and BC5 (GCA_041344855.1, GCA_040109495.1, and GCA_014654865.1, respectively) studied by Croatti et al. (2026). The RL30 genome assembly and annotation generated in this study were used as a reference.

Genes within the CWPS locus were identified by extracting GFF3 entries corresponding to the locus tag range of interest (ENBNMP_11300 – ENBNMP_11390) from *L. crispatus* RL30. Nucleotide sequences of these CDS were extracted using gffread with the corresponding FASTA file. Separated nucleotide BLAST databases were constructed from the assembled genome of each strain (CTV-05, BC1, BC3 and BC5) using makeblastdb (BLAST+ package). The CWPS locus query sequences were blasted against each strain's database using blastn. BLAST results for each strain were filtered for high-quality hits (>95% identity; alignment length > 50 bp, E-value < 1e-5). The filtered results are summarised in Table S4.

Table S4. Presence (+) and absence (−) of CWPS locus genes in selected *L. crispatus* strains. Genes are listed in their order in the RL30 locus (ENBNMP_11300–ENBNMP_11390). A gene was scored as present when a BLASTn hit met the filtering criteria given above (>95% identity, alignment length >50 bp, E-value <1e-5). S11, RL30_S11.

Annotation	BC1	BC3	BC5	CTV-05	RL30	S11
IS256 family transposase	+	+	−	+	+	+
Hypothetical protein	−	−	−	+	+	−
Polysaccharide biosynthesis protein C-terminal domain-containing protein / flippase (Wzx)	−	−	+	+	+	−

Annotation	BC1	BC3	BC5	CTV-05	RL30	S11
UDP-galactopyranose mutase	–	–	+	+	+	–
**Glycosyltransferase group 2 family protein / GT3**	–	–	–	+	+	–
IS256 family transposase	+	+	–	+	+	+
**– / GT2**	–	+	–	+	+	+
IS110 family transposase	–	+	+	+	+	+
**Glycosyltransferase 2-like domain-containing protein / GT1**	–	+	–	+	+	+
Glycosyl transferase family 1 domain-containing protein / GTB	–	+	+	+	+	+
Glycosyltransferase 2-like domain-containing protein / GTA	–	+	+	+	+	+
Oligosaccharide repeat unit polymerase (Wzy)	–	+	–	+	+	+
IS256 family transposase	+	+	–	+	+	+
Hypothetical protein	–	+	–	+	+	+
DUF4422 domain-containing protein	+	+	+	+	+	+
Putative sugar transferase EpsL	+	+	+	+	+	+
Tyrosine-protein phosphatase (CpsB)	+	+	+	+	+	+
Tyrosine-protein kinase (CpsD)	+	+	+	+	+	+
Capsular polysaccharide biosynthesis protein (CpsC)	+	+	+	+	+	+
Transcriptional regulator	+	+	+	+	+	+

Additionally, the priming glycosyltransferase gene *epsE* (Accession: N506_0400), as mentioned in Croatti et al., (2026), was independently queried against *L. crispatus* RL30 and derived isolates, showing >70% nucleotide identity to a gene annotated in our dataset as a putative sugar transferase (*epsL*). Only a single hit was detected per genome, indicating that this correspondence likely represents the same genomic locus.

Query sequences

The nucleotide sequences below are the 20 CDS of the *L. crispatus* RL30 CWPS locus (ENBNMP_11300–ENBNMP_11390) that were used as BLAST queries. They were extracted with gffread from the RL30 genome assembly deposited in the European Nucleotide Archive under project accession PRJEB106179.

```
>ENBNMP_11300|IS256_family_transposase

ATGAATGATTTTACCAAAGATTTTGCTCAAGCTCTATTCAATCCAGACAAAATAAATGATTTATTGCGCAAAGAGCT
ACAACAGGCTGTTAATAACTTGTTAGAAGCTGAATTGACCGCCTTTCTGGGCTATGATCCCTATGCCAGAAATGGCT
GGAATACTGGCAACTCTAGAAATGGTGCTTATTTCCGCAAAGTTGATACGCAATTTGGCCCAATTGAAGTGCAAGTG
CCCCGGGATCGCAACGGTCAATTTCATCAGCACACGCTGCCTGACTACAAGCAGCACTCGGATATCTTGGAAAGCAT
```

GATTATCAAGCTTTACTCCAAGGGCGTGACCACCAGGGAAATAGCCGATCTAATTGAAAAGATGTATGGCAGCCATT
ACAGTCCAGCTCAGGTATCAAATATTTCCAAGCAGATGATTCCAAAGGTTGAGGCTTACCACAAACGCAAGCTAAGC
GACAAGTTTTTCTGCGTCTATTTGGATGCGACATACCTTCCTTTGCGCCGAGAAACATTTGAGCGTGAAGCAGTCTA
TATTGCCATTGGCATCAAGCCTAATGGGCACAAGGAGGTCATTGACTACTGCATTGCGCCTAGCGAGAACATTGAAG
TCTGGACAGATATGCTTCAAAACATGAAATCCCGAGGCTTAAAGCAAGTTGAGCTTTTTCTTTCTGATGGTGTTGTT
GGCATGAAAACAGCCTTGGCCAGGACTTATCCTAAAGCTCATTTTCAACGCTGTTTGGTTCATGTCATGCGCAATAT
CTGCGCTAAAGTACGCGTCGACGATCGTGAAAAGATCATGAACGAATTCAAACAGGTGCATCAACAGACAAACAAAG
AAGAAGCCACAGCTGTCCTGCATGACTTCTATACCAAATGGGGCAAGGTTTACAGCCATGTTATCAGAAGCTTGAAA
GACATAGAACCAGATCTGCTGGTCTTTTATAACTATCCCAAGCAGATCAGAGCTTCAATTTATTCGACTAACATGAT
TGAATCATTTAACAATGTGATCAAGCGCAAAGCTAAGCCTAAGGCAGAGTTTCCAACTGAACAGTCGCTTGATGCTT
TTATTGGTATTCAGGCAATGAGCTACAATGACCGTTATTTCAATCGAATTCATAAAGGCTTTGGTCAGGTTCAGGAC
ACCTTAGAATCCTACTTTGATTAA

>ENBNMP_11305|hypothetical_protein

ATGAAAAGCTTTTTTCAAAATAAAGTTAATGGACTTATAGTAGCTATATCAACTGTATATACAGCTATTTTAATTAA
CGTATATTTATTTTTAACTTCTGAAATTGAGGCGCCTGGATATATGATATATATTTTTATCATTGTCGTTTTAATGG
TAAGTATGTGCTTTTTATCAGATTATAGGGTAAAATTAGCTGTTTTTATAAATAGATATCCTGCAAAATTAGTGAAT
ATAATGAGTGCATGTATAATAATAGCAGCCAGTATTTTTAGTTTTTTGCTTTTAACTGCTGATAAATCTTCTTCTCC
CAGAACAACAGTAATGTATCATGGTCTAATAAGTTTATTGAGCTTTGCTTTAATACCAGTTTTAAATAGATTATCCC
ATGAACCAATTGTAGATGTGAAAATACTTACTAAAAATGTAATTAATAATTCTGAAAAAATTGTTTTCTCTTCAAAA
GATGAGTTTAAACTAACAGCTCTTTGGAATGGTAATACTGAAGAAGTAATCCAATTTAGAGGCTTTTGCTTACAAAA
TGACGAAAATAAATTGCATTCTTATGATGTACCAAAAGATAATATTGTTTTTATGCCAATTCATAAGACCAGGTCAA
GAGAGAAAGTTTCTATAGCTTCAGTACCTGAACAAGTGAAGCCATATTCATTGGGTAGAGAATATAATATAAGAATT
AAAGATATTTTAACAGCTTTAAAAAGTAGAAAAGTAGAGACTGTAAAATAG

>ENBNMP_11310|Polysaccharide_biosynthesis_protein_C-terminal_domain-
containing_protein (flippase)

ATGAAGGTCATTCGAAATTATCTCTATAATGTAGGTTATCAAGTATTAGCAATAATAGTTCCGCTTATTACATCGGC
ATATGTCAGTCGTGTATTACGACCTGAAGGTGTAGGTGCTAATGCTTTTACAAATTCCATTATTCAATACTTTATCT
TATTTGCTAGTATGGGAATTGGTTATTATGGGAATCGGCAAATAGCCTATGTTCGAGATAATCGAACTAAAATGGCC
AAAACTTTTTGGGAGATTCAAATAGTTAAAACAATAATGACTCTTGTATCAATTATTGCTTTTGAAATCTTCCTTAT
TTTTTATACTCGACAGTTTGATTATATGTTGGCTCAGTCGCTTAATCTAATTGCTGTGGCTTTTGACATTTCTTGGT
TTTATGAAGGTGTTGAAAACTTTAAAGTAACGGTTTTAAAGAACTCACTGGTAAAGATTGTATCAATGATAGCGATC
TTCGTTTTTATCAAGGGACCCTATGATGTAACTTTATATATTGTAGTATTGGCATTGTCAACTCTACTAGGTAACTT
AACTTTGTGGCCGGATATTTATCGAGATTTGCCTAAGGTTAAGATTAGGAGTTTAAATCCTTGGATTCACTTTTTAC
CAATGGTTGAGCTTTTCATACCGCAAATAGCTACTCAAGTTTATGTACAATTGAATCGTACGATGTTGGGCGTCATG
GTTAATGAAACAGCATCTGGTTACTATCAATATTCTGATAGCTTAGTGAAGTTGATTTTGGCTTTAGTGACAGCAAC
TGGTACAGTCATGTTGCCACATGTAGCCAATGCGGTATCACATGGTGACATGAAGAAGGTAAATCAGATGTTGTATA
AGTCTTTTGATTTTGTATCGGCTATGGCTGTACCAATGATGTTTGGCTTGGCAGCAATCTCCTTAACATTAGCTCCA
AAATATTATGGTCCCGGCTATGGTCCAGTAGGTCCAGCAATGATGATTGAGTCTATCGTGATTTTGATGATTGCTTG
GAGTAACGCACTTGGCGTGCAGTACCTATTACCAATTCACCATCAGAAGCAATTTACAATTTCTGTTACAGTGGGTG
CCGTAGTAAACATAATTTTAAATTTCCCGTTGATTCACTTTTATGGTTTGAATGGTGCCATGTGGTCAACTGTTTTG
TCCGAAATTAGTGTTACTTTGTATCAACTGATTGCGGTTCGTGGTTTGTTGAGCTATAGGACTCTATTTAACAGCCT
ATGGAAGTATTTGGTCTCTGGTGTAGTAATGTTTTTGCCAGTTTTTTGGATGAATACACACTTGAAAGATTCTTGGC
TAATGATGGGACTCGAAGTTATTGTTGGTGTTGTCGGTGTTATGGTGATGCTGCTAAGAGCGCCTATTGTGGATGAG
GCTAAGAAGTTGATTCGGGAGAAGTTGGGAAAGTAA

>ENBNMP_11315|UDP-galactopyranose_mutase

ATGGAAAATAATTATCTAATAATTGGTTCTGGTCTTTTTGGAGCAGTATTTGCTCATGAAGTAGCAAAACGTGGAAA
CAAAGTTACAGTAATTGAAAAAAGAGATCATATTGCAGGTAATATTTATACTAAAGAAGTAGATGGGATTCAGGTTC
ATCAATATGGTGCTCACATTTTTCATACTTCAAACAAAGAAGTTTGGAATTATGTACAACAATTTGCTGAATTTAAT
CGTTATACTAATAGTCCTGTTGCCAACTATAAAGGACGTATGTATAATCTGCCATTTAATATGAATACTTTTAGCGA
GATGTGGGGAGTAGGGACTCCGCAAGAAGCTATGGCTAAGATCAATGAACAACGACAAGAAATGGCAGGCAAGGAAC
CACAAAACCTTGAGGAACAAGCTATTTCTTTAATTGGACGTGACATCTATGAAAAGTTGATCAAGGGTTATACAGAA

AAGCAGTGGGGCAGACCATGTACTGAATTGCCTGCTTTTATTATCAAGCGTCTTCCAGTGCGTTTGATTTACGATAA
CAATTACTTCAATGATGATTACCAAGGTATTCCTATTGGTGGTTACACAAAAATGGTGGAAAAGATGCTTGATCATC
CAAATATTGAAGTTAAATTAAATACTGATTTTTTTGATGATAAAGAAGAGTATCTTAATAACTATGATAAAGTTGTC
TACACCGGTATGATTGATAAGTTCTTTGACTATAAATTAGGTGAACTTGAATATCGTTCACTTCGTTTTGAAACTGA
AGAAAAGAATGTTGGCAACTATCAAGGCAACGCAGTGATCAACTATACTGAAGCTGAAATCCCCTACACTCGTATAA
TTGAACACAAGCATTTTGAATTCGGTAAGGGTGATCCAGATAAGACGATTATTACCCGGGAATATCCAGCGGATTGG
AAGCGTGGAGACGAGCCATATTACCCAGTGAATAATGATAGAAATAATAGTTTGTACGCTAAATATGCTGAATTAGC
AAAGAAACAAGACAAGGTAATCTTTGGTGGTCGTCTTGGTCAATACAAGTATTACAATATGGATCAGGTAATTGATG
CTGCACTCGATGCGGTAAATAATGAATTTGGTGAATAG

>ENBNMP_11320|Glycosyltransferase_group_2_family_protein (GT3)

ATGTCAGCAGTTTACGAATTACAAGATATAGAGATAAATACAGTCCCTTATTTTGAAAATAGCATGATAAAAAAAAC
GTTTTTTAAATTCTTTATGAATAGAAAAACAGCTAACTTTACTGCAAAAATGTATGTTACTCTAAAAAAATAG

>ENBNMP_11325|IS256_family_transposase

ATGATTGGTTTGAACAAAAAAAACACATACAGAAAGAAGAACTCCTTCATGAATGATTTTACCAAAGATTTTGCTCA
AGCTCTATTCAATCCAGACAAAATAAATGATTTATTGCGCAAAGAACTACAACAGGCTGTTAATAACTTGCTAGAAG
CTGAATTGACTGCCTTTCTGGGCTATGATCCCTATGCCAGAAATGGCTGGAATACTGGCAACTCTAGAAATGGTGCT
TATTTCCGCAAAGTTGATACGCAATTTGGCCCAATTGAAGTGCAAGTGCCTCGGGATCGCAACGGTCAATTTCATCA
GCACACGCTGCCTGACTACAAGCAGCACTCGGATATCTTGGAAAGCATGATTATCAAGCTTTACTCCAAGGGCGTGA
CCACCAGGGAAATAGCCGATCTAATTGAAAAGATGTATGGCAGCCATTACAGTCCAGCTCAGGTATCAAATATTTCC
AAGCAGATGATTCCAAAGGTTGAGGCTTACCACAAACGCAAGCTAAGCGACAAGTTTTTCTGCGTCTATTTGGATGC
GACATACCTTCCTTTGCGCCGAGAAACATTTGAGCGTGAAGCAGTCTATATTGCCATTGGCATCAAGCCTAATGGGC
ACAAGGAGGTCATTGACTACTGCATTGCGCCTAGCGAGAACATTGAAGTCTGGACAGATATGCTTCAAAACATGAAA
TCCCGAGGCTTAAAGCAAGTTGAGCTTTTTCTTTCTGATGGTGTTGTTGGCATGAAAACAGCCTTAGCTCAGACTTA
TCCAAAAGCTCATTTTCAACGCTGTTTGGTTCATGTCATGCGCAATATCTGCGCTAAAGTACGCGTCGACGATCGTG
AAAAGATCATGAACGAATTCAAACAGGTGCATCAACAGACAAACAAAGAAGAAGCCACAGCTGTCCTGCATGACTTC
TATACCAAATGGGGCAAGGTTTACAGCCATGTTATCAGAAGCTTGAAAGACATAGAACCAGATCTGCTGGTCTTTTA
TAACTATCCCAAGCAGATCAGAGCTTCAATTTATTCGACTAACATGATTGAATCATTTAACAATGTGATCAAGCGCA
AAGCTAAGCCTAAGGCAGAGTTTCCAACTGAACAGTCGCTTGATGCATTTATTGGCATCCAGGCAATGAGCTACAAT
GACCGTTATTTCAATCGAATTCATAAAGGCTTTGGTCAGGTTCAGGACACCTTAGAATCCTACTTTGATTAA

>ENBNMP_11326|GT2

TTTCTTTTTTTCAAGTTTCACCGCATGCAAAACTTTATAAAACCCAATTGCTAAAAAATGTTAGTTTCCCTAAACAC
GTGAGTTATACGGATTTTTTGTTATATTTGGTTGCGTTGTCTAGAGCGAAGAAAGTATCATATTATGATAAAGCCCT
CGCTTTTTATTTACAAGATAGACCAGGAAATACAGCAACTGATATAAGAAAGTCTATAATTAATGATTATTTAA

>ENBNMP_11330|IS110_family_transposase

ATGGAATCACAAATTATTTTCGGTATTGATGTTAGCAGTAAATCTTCCACAGTTTGTGTGGTTAATGACCGGATCAA
ACAAGGCGAATCTTTTAGAATCTCTAATGATTCCTTTGGCTATCAAAAACTTTATGAGCAACTGAACCAATATTTGA
TAACTCCTTTAGTTGTTTTCGAAGCTACTGGTGTTTATTCTCTAAGCCTGCAAGCTTTCTTAGAGGATTATCATATT
AAGTATTTGAAGCTTAATCCGCTTAAAGCGAAAAAGCTAATGGACAATAATTTACGCCATAATAAAACTGATAAGGT
AGATGCTTATCGCCTGGCTTTAATTCAATTTAATGCCCCGCAAAAGCTGCGTGATCCACAGCCCAGAGAGTATCATG
AACTTCAGAATGCCAGTCGCTATTATGAAGAACTTACTAGGAGTATAGTTACTACTAAAAATCAATTGCACCGCAAC
CTCCAATCCACTTTCCCACAAATTGAAGAAATACTGTCTCATCCATCTGGCAGAATTTATTGGGCGCTTGTTAGTCT
TTTCCCACATGCTGGATATGTTCTAGAAAAGAATGAGACACAGGTAACTCAAATGCTTAATTCTATTTCTGGAATAG
GACAACAAAGGGCACGGTACTTAACCACAAGATTATACGCTTTTGCTAGAGAAGCTTATCCTTATGATTCCAAAGAT
AGTATTATCGTTAGAGCTATTCAAAGGAACATTTCTAATTTATTTTCACTCCAGCAAGAACGCGACTATGTAATTAA
CTACATGGAAAAGCTGGCTAAAGCAGTAAACGCTCGTGCTTTATCCATCTATTTGAGTATCCCAGGGGTTGCTAGAA
TTACAGCAGTGAGATTAGTAGCAGAGCTTGGTGATCTAAGACGGTTTAGCACTTCAGCTCAAATTGATGCGTTTGTT
GGCATTGATCCAGGAAGGTACCAATCTGGTGAAAAAGACAGTTCTCTTGGCATTACCAAGCATGGCAATCATATTGC
CAGAAAAATACTTTATCGAGTTATTACTCAAATGGAGACGGTAAAGGCTACGCAGCCTTGCCATATAACTGATTATT
ATGACAAGAAAAAAACGATCTTCAAATAGACAAGGCTATAAGAAAATCGCCATTGCATCAGTCCATAAACTGATACGC
ACAATGTTTGCTCTTATCAAACATGATCAATTATATGATTACAACATAGCCACCGAAAATAAAAGACTTTAG

>ENBNMP_11335|Glycosyltransferase_2-like_domain-containing_protein (GT1)

ATGAAATCAGACATAACCGTAATTGTTCCAATGTATAATGTTGAAAAATACGTAAAAAAATGTTTTGAGTCATTAGA
AAATCAGACATACAGTAATTTTGAAGTTTTGGCTATTAATGATGGATCGCCAGATAATTCTAGTAAGTATGCGAAAG
AATGTGCTGCGAAAGATAATAGAATTAAAGTAATTGATAAAGAAAATGGTGGCTATGGTTCAGCGTTAGAGTTAGCT
ATAAATAGTATTAATTCTGATTACTTTTTGGTGTGTGATCCTGATGATTGGTTGGAAGAAGATTGTTTAGAAGTACT
TTATACATTTGCCAAAAATAATGACACTGATATTGTAGTAGGGGATCGATTTGATGTGTATGCTGATGATGGAAGTG
CTCATAAATGTTCTGTGAAACCAGGATATTTAGAAAGTATTGAACCTAAAAAAGTATATTCAGAAACACAGGATATA
CAGCTGTTTTTCTACCCAAAGTCAAGTATTATCAATGATTTAAGAAAATAA

>ENBNMP_11340|Glycosyl_transferase_family_1_domain-containing_protein (GTB)

ATGAAAAAGATTTTAGAGGCGTTTGGTGAGCCAATTTTGTATGGCGGGCAAGAGGCTTTTGTATTTAGAACAATTGA
AAATATGAACAAAAAAGGTCTAAAATTCGATTTTTTAACACCATATTATGCTGATAATCCCGACTATATTAGCTTTA
TTAAATCACTAAATTCTACGCTATACTCTTTTAATTTACCTTTTAATGTTGGAAAGAATAGATTTAATGTTGTAAAT
GCATATAAAAAAATTCTGCTAGATAATCAATATGATGTTGTGCATATAAATAGTGGAAGTATATCTATACTTGCATT
ATTTACCTTGTATGCTAAAAAAGCAGGTGTGAAAAAAGTTATTGTGCATTCGCACATGTCAGGAAAAAAATAAAAATG
TAAAGCATGAAGTAATTAAAAAGATTTATGCACCAATCTTTAGTAAATATGCTGACGTTTTGGTTGCTCCAACAAAA
AAAGCTGCATATTGGCAATTTTCAAAAAAAATTTTTAACAAAAAAGGAAGAATTTTGAAAAATGGAATTAACATACA
GCAGTATGCATATAATATTGGGACTAGAGAACATTATCGTAACAAATTGAATATTTCAGATCATGAAATACTAATTG
GGCATGTTGGAAGGCTATCACCTGAGAAAAATCAAATATTTTTAATAAAATTATTAAGTTATTTTATTAAAAATAAT
ACTAAGGCTAAGCTGTTACTTATAGGAGCTGGTCCACAGGAAAAATCAATACGAATGAGTATTAAGCAAAATCGATT
AGAGAAATATGTTCAATTAATAGGAAATGTAGATAATGTTGAAGATTATCTACAAGCAATGGATTTATTTATTTTTC
CTTCTGAATATGAAGGACTAGGAATTGCAAGTATTGAGGCTCAGGATGCTGGGCTTCCGGTATTAGCTTCCACTAAT
GTTCCTCTAGATATTAAGGTTACTGATAACGTGATGTTTCTTGACTTAAATTTATCGCTTAAAGAGTGGTATAAGCA
AAGTTTAAAGTTACTATCCAGGAAGTATGATCGAGCAGAAAATACAAATTTTTTAAAAAAACAAGGATACGATATTA
GAAATACTGCTGAAAAGTTGAAGAAAATTTATTTGGGGTAA

>ENBNMP_11345|Glycosyltransferase_2-like_domain-containing_protein (GTA)

ATGAGGAAACAGCAAGTTGCCGCTATTGTAGTAACATATAATAGAAAAAAGTTACTAAAAGAATGTATTAAAAGCTT
ATTGGAACAAACCGCAAAGCCAGATATATATATTATTGATAATAATAGTACAGATGGTACAGTTAATTATATTGCAA
AATATATTGATGATAACAGTATAAACTATGTTAATACGGGGAAAAATCTTGGTGGTGCTGGTGGATTTCAGTATGGT
ATAAAGATGGTAGCTCAAAAGGATTATGATTTTGTTTGGATTATGGATGATGATTGCATACCAACTAAAAATGCTTT
GAATGAGTTTCTTAAGGCAGATATAAAACTAAATCATGATTATGGTTTTTTGTCTAGTAAAACTTTGTGGACCGATA
ATACCTTGTGTACGATGAATATTCAGAGAAGGACATTAACAAAAAATGTGTCTGATTTTTCATCTGAATTGATACCT
GTTACTTTAGCTTCTTTTGTTTCCTTATTTGTTCCAGTTTCAGTTATCAAAAAAGTAGGGTTACCAATTAAGGAATT
CTTTATTTGGACTGATGATTGGGAATATACAAGACGAATTTCCTCAAAATATGAATGTTATGTTGTAACTAATAGTA
TTGTTGTACATAAAACAAAAACTAATGTTGGTGCAAACATTGCAACAGATGTCCCAGAAAGAATAGAAAGATATAGA
TATTTGTATAGGAATGATTTTTTCTTTTATAGAAAGGAAGGGCTAAAAGGATATGGATATGAATTTTTACGTTTAAA
TGATCACATGATAAGGGTTTTGTTCAAAGCTAAAAATAATAAACGTAAAAGGTTAAAGATGATATTTAAAGGAACAA
TTGAGGGATTACGTTTTAATCCGTCTATAGAGTATATAAAATAA

>ENBNMP_11350|Oligosaccharide_repeat_unit_polymerase_Wzy

ATGTTTAAAGAAATAAATTTAAGGAATCATTCATCCTTACACAAACTATTTTACAGTCTCAGATATGTATCTATTTA
TATAGGCGTTTTTATTTTTATGTTTGGGCTACAGTTATATAGGCTAAATAGTTTTTCCTTATCTGGGTACACAATTA
AAGATGCTTTAATTCATAATAGATATTATGCGTATATGTTGTTAACGTTTCCGATGACGGAGGTGCTAGCGACTGCT
AAACGAAGAATGAAATTTATTAATAATATATATTATATGGGGGTTGCTATATTAATATTTAGATTTAGCGCGTGGTT
TTTGTACAATAAATTACGTATGAATGTAGCTCCAGGTTATTTTGAAGTAATGGGATTTGCTTGGGCGAGAAATGGAG
TAAGTAGATTACCAGGTACTTTTTTAGATAATTACGTATGGGTTATGGCTCTTTTCAAAATCATCAAAGAAAAAAGT
GTTGTAATTAAAACACGTAATATCTCAGTTATAGTATTATGCTTTTTATATGCATATATTGTGTATGACTCACGTTC
ACAACAAATAGCCTATTTATTAAGTTTAGTGGTATTCATACTTATCGTTAGTGATACTTTTAAAAGTAAAGCTCTAG
ATGTGGTAGGAGCTATAGCCATTAGTCCAATTATTTTTAGAGGAAATTACTTAAATAAATTGGTTAATACTTTTTCA
ACTTCAAATGCAGACTATGGATCTAGTACTCAAATAAGAATGATGACATTAAATACATATCAAACTCTTTGGATAGA
AAGAGGTGTATGGTGGGATATGGTATATCAAACGATGGGAATTATTTTAATATATTGGATACACATATGGTAAACC
AATCAGATTTAGGTATATTATCTATGTTATTTCAATATGGAATAATAGGTTTTGCTATTTTTATATCGCCATTTGTA
ATGGGATTATATGTAAGCTTAAAAAAAGTCAAATTTGTTCAAAATAAGTTTTTGTTTTTACTAACTTGCACTACTGC

ATTAACTAGTATAATGTCACAAAATATGTATGATCCATTTAGATTTCTAATAGTACCATTTTTATTAAGTTTTATTT
CTGTAGTTAGCTTTATGAATAAAAAGGAGAATTAA

>ENBNMP_11355|IS256_family_transposase

ATGATTGGTTTGAACAAAAAAAACACATACAGAAAGAAGAACTCCTTCATGAATGATTTTACCAAAGATTTTGCTCA
AGCTCTATTCAATCCAGACAAAATAAATGATTTATTGCGCAAAGAACTACAACAGGCTGTTAATAACTTGCTAGAAG
CTGAATTGACTGCCTTTCTGGGCTATGATCCCTATGCCAGAAATGGCTGGAATACTGGCAACTCTAGAAATGGTGCT
TATTTCCGCAAAGTTGATACGCAATTTGGCCCAATTGAAGTGCAAGTGCCTCGGGATCGCAACGGTCAATTTCATCA
GCACACGCTGCCTGACTACAAGCAGCACTCGGATATCTTGGAAAGCATGATTATCAAGCTTTACTCCAAGGGCGTGA
CCACCAGGGAAATAGCCGATCTAATTGAAAAGATGTATGGCAGCCATTACAGTCCAGCTCAGGTATCAAATATTTCC
AAGCAGATGATTCCAAAGGTTGAGGCTTACCACAAACGCAAGCTAAGCGACAAGTTTTTCTGCGTCTATTTGGATGC
GACATACCTTCCTTTGCGCCGAGAAACGTTTGAGCGTGAAGCAGTCTATATTGCCATTGGCATTAAACCTAATGGAC
ATAAGGAAGTCATTGACTACTGCATTGCTCCTAGTGAGAACATTGAAGTCTGGACAGAGATGCTTCAAAACATGAAG
TCCAGAGGCTTGAAGCAAGTTGAACTCTTTCTTTCTGATGGTGTTGTTGGCATGAAAACAGCCTTGGCCAGGACTTA
TCCTAAAGCTCATTTTCAACGCTGTTTGGTTCATGTCATGCGCAATATCTGCGCTAAAGTACGCGTCGACGATCGTG
AAAAGATCATGAACGAATTCAAACAGGTGCATCAACAGACAAACAAAGAAGAAGCCACAGCTGTCCTGCATGACTTC
TATACCAAATGGGGCAAGGTTTACAGCCATGTTATCAGAAGCTTGAAAGACATAGAACCAGATCTGCTGGTCTTTTA
TAACTATCCCAAGCAGATCAGAGCTTCAATTTATTCGACTAACATGATTGAATCATTTAACAATGTGATCAAGCGCA
AAGCTAAGCCTAAGGCAGAGTTTCCAACTGAACAGTCGCTTGATGCTTTTATTGGTATTCAGGCAATGAGCTACAAT
GACCGTTATTTCAATCGAATTCATAAAGGCTTTGGTCAGGTTCAGGACACCTTAGAATCCTACTTTGATTAA

>ENBNMP_11360|hypothetical_protein

ATGAGATGGAAAACAGTAAAACTGTATGAATATTCAATTTACCTTATAGTTTTTTTATATTTAGGTTTTATCAACTT
TTTTAAAGACTTAAATTCTTTAAGTATTAGTATAGGTGTTGTTATATCTGTTTTACTGTATTTTATTTATGTAAATC
AAAAAAATAGAGAAATAAAAGTTTCAAGATATGTGAAACTGTCAAATCTTTTGTGTAAATAG

>ENBNMP_11365|DUF4422_domain-containing_protein

TTGAAAATAGAAATTTTGGTTGCTGCGCATAAAGAGTTTCCTATGCCCAAAAAGGAAGGATATTTACCAGTATTAGT
AGGCGCAGTAAAGAATTATAAACCAGGTATTTCCTATCAGCGTGATGATGATGGTGAAAATATATCCTCTAAAAATC
CTAATTACAATGAACTAACTGCTGTTTATTGGGCTTGGAAGAATTTAAAAGATGTTGACGCTATTGGATTAGTTCAC
TATAGAAGATTACTCTTTGAGAAAAGACCATATTCTTTAGATAATGTAATTAGTATTGAGAAAGTAAATCAGCTTTT
ACAAAAATATGATGTAATTTTACCTAAAAAAAGAAATTATTATATTGAAACTAATTATTCACATTACATTCATGCAC
ATCATAAAGAGCCTTTGGATGAAGCAAGAAAGGTAATTGCTGAAGTATATCCAAATTATCTTCCTGCTTTTGATAAA
GTTATGAAAAAGCGTGGGGCTCATATGTTTAATATGTTTATTATGAAAAGGAATGCATTTGAATCATATTGTAGCTT
CATGTTTGATGTTCTAAGTAAGCTAGAAAATAGCATTAATATTTCTGAATATTCTGTGCAAGAAGCGAGAGTATTTG
GATATATTTCTGAGTTGTTAATGGATGTTTGGCTTGGTACCAATTCATTTACTTATGTAGAAGTTCCTTGGGGACAA
ATTGGTGGAAAGAATACTTTAAAGAAAGGCTTTTCTTTAATTAAACGAAAAATAGGTATAAAAACGAAAACACATTT
TTAA

>ENBNMP_11370|Putative_sugar_transferase_EpsL

GTGTATCACACGATTAAGGGGATATTTGATATACTAGCTAGCGCACTTGGGTTAATTTTACTATCTCCGTTATTTTT
ATTTTTGATAATAAAAATAAGACATGAAGATGGTGGTCCTGCATTTTATTCTCAAGAACGAATCGGTAAAGATGAGA
AGCCGTTCAAGATGTGGAAGTTCCGTTCAATGGTCGTTAACGCTGATCAAATGCTTGATGAACTAGAAGATCAGAAC
GAAATTGACGGCGCAATGTTCAAGATCAAGGACGATCCAAGAATTACGAAGATCGGACATACAATTCGCAAGTATAG
TCTTGATGAATTGCCACAACTGTGGAATGTCTTGATCGGCGATATGTCACTAGTCGGTCCACGGCCCCCTCTACCCT
CTGAAGTAGCTGAGTATACTGACTACGATAAGCAACGTTTGCTCGTTATGCCAGGTTGTACTGGCTTGTGGCAGGTA
ACACGCCGTAGTGAAGCTGACTTTGACGAAATGGTTTGGCTTGATATTGTGTATATCAACCATTCCGGTTTGTGGGA
AGATTTGAAGTTGATTGTTAAGACAATTGGGGTGGTTATTCATCCTAATGGAGCGTATTGA

>ENBNMP_11375|Tyrosine-protein_phosphatase

ATGGTTTTAGTCGATATTCACTGCCACATCTTGCCTGGCATTGATGATGGCTCAAAAAATTGGGACACTTCAATTAA
GTTAGCCAAGGCTGCGGTCAAAGATGGCGTAACGCATGCAATTTGTACGCCGCATACACTGAATGGTCGTTACACTA
ACCATAAGGATGATATCGTCTGGCTGACTGATCTCTACCAACAGAAGCTAGATGAGGCCAAGGTGCCGTTGACGGTT

TTCCCAGGGCAGGAAGTTCGCTTGTCGGGTGACTTAATTGATGCGCTTGATAACGATGATATCCTGTTCTGCGATGA
AGACGGTACTTATATGCTGTTGGAATTTCCAAGTGAGGACGTGCCAACTTATGCGCAAGACACGATCTTCAAGATCA
TGCAGCGCGGGGTGACGCCGATTATCGTACACCCAGAGCGCAATAGTCGCATCTTGAAGGAACCGGAAATTTTGCAA
GGGATGCTGGAACAAGGCTGCTTGGTGCAAATTACTGCTAGCTCTTATACAGGAATCTTTGGCAAGAAAATCGAAGA
GATGTCGCGTAAGTTGATTGCGACGGGGCAGGGATGCACCTTTGCTAGCGACGCACACGACTTGCCAAGACGGCAGT
ATCAATTGAGTGAAGCTTATAAAAAGATGAGTCAAGAATTCAGTCAAGATCTAGCTCAGCAGTGGCAAGATAATGCT
AGAAGTATCATCAATGGCGACCCAGTTCAGATGGATTGGCACCCATTGAAGCAGAAGAAAAAGTTCTGGTTATTTTA
A

>ENBNMP_11380|Tyrosine-protein_kinase_CpsD

ATGGCATTATTTAGAAAAAAGCGTGGTACCGACGATACAATTAAGCATGGTGCCAAATTAATCACAGTTGCTGATCC
TCGTAGTGCGGTATCTGAACAATTCAGAACTATCCGGACTAACATCAACTTTATGGCAGTTGATGAAGAAATTAGTA
CCTTAGCTTTTACTTCAGCTAACATCAGTGAAGGTAAGTCAACCGTTACGGCCAACGTAGCGATCACCTATGCACAA
GCAGGTCGCAAGACCTTGCTAATCGATGCCGACTTGCGTCGTCCAACTTTGCACAGTACGTTCAACGTTAAGAACAA
TACTGGTTTGACAACTGTTCTTACTTCAGAAGCTGACGCAATCAATTTAAATGATGTGGTTGAAGAAAGTGGAATTG
ATAACTTGTCAATCTTGACTTCTGGTCCGATTCCACCAAACCCAGCTGAATTGATTGGTTCACGCAGAATGGAAACC
TTCATTGAATTGGTTAAGTCACATTATGATATGGTTATCATTGACTTAGCTCCTGTTCTTGAAGTATCCGATACGCA
AGAGTTGGCTAGTCACCTTGACGGCGTAGTCTTAGTAGTACGTCAAGGCGTTACGCAAAAGGCTGGGATTACTAGAG
CAGTGCAAATGTTAAAATTTGCCAAAGCTCGGATTCTAGGCTACGTCATGAACGACATTAGAGCTGAAAACGGTGGC
TATGGTTACGGCTACGGGTATGGCTATGGTTATGGATATGGCGCTGAGAAGAAAAAGGGCCTGTTCGGTAGAAAAAA
GAGTGACGATCAATAA

>ENBNMP_11385|Capsular_polysaccharide_biosynthesis_protein_CpsC

ATGGAACAAGAGCAAAAGCAAACTGAAAATACAATTGATCTTACTCAATTGCTACAAATTTGCCGTCGCCACATCTG
GGCACTGATTATCTGGAGTGTTGGTTTGGCTTTAGTCGGCTGGGGCGTCGCTAATTTTATTATTTCACCAAAGTATA
CTTCTAATGCACAGATCTTGGTTAACCAAAAGGCTAACAAGAATGACCCGAATGCGGCATATAATACTCAACAAGCC
AACATGCAAATGGTTACTACCTATAAGGATATTGTAACTAGTCATGTTATTTTGCAAGATGCTTCTAATCGTTTAGC
TAACCCAGTTAGAGTGGTTAAAAAGGCTAAACCTGCTAAGTACAAGACTAATGCAGAGGGTCGCCGAGTATTAGTAA
GAAAGGCTCAACCAGAAGTAGTTGAACGTAGCGGTAAGAGTTACAGCGTTTCTACTAGCGAATTAGCCAAGAGCGTT
TCAGTTAATACTCAACAACAATCACAGGTCTTCTCAATTTCTGCTACTGCAGATACACCTGAAAAGGCAAAGGTTGA
AGCTAACGCTGTAGCGCGTTCATTCAGAGATCAAATTCCTAACATCATGAACATTAACAACGTTACGATCGTTGCGC
CAGCAACTGATGGTAATCAATCATCACCTAATGTGAAACTCTTTACCTTGGCTGGCTTTGTTATTGGCTTGGTTTTA
AGTTTCGCAGTAGTCTTAATTCGTGAAATGTCCGACACTACAGTTAAGGATGATGCATTCTTGACTGATAACTTAGG
TTTAGTTGACTTAGGTCAAGTTTCACACTTCCATGTGTCATCATCATTTGTCATTAGAAAAAAGAATAAGAATTCTA
ATGGCCCTAAGCGTAGAAGCCGTCGAGTATAG

>ENBNMP_11390|Transcriptional_regulator

ATGGAAAATAACGCAGAATCAAGTAATAAACCGATACATTGTCATCGCCATCATCACCACCGTCGTCACCGTAAGTT
CTGGCGCGGTTTTTGGATTATTATTGGCGTGATCATTGTAGTCGGTCTTTTTGTTTGCGGGATGATCTACAAAAATT
TGCGTGATACGACGCAGAATATGTACACTCCCGTTGCTAAGACTACCAAGAGTAATAAAGGACGTACTCTTGATAAT
TTACTGAATGAGAAAAAGCCAATTAATATCCTGTTATTGGGTACAGATACTGGTGCCATGGGTCGTCACTGGAAGGG
TAGAACTGATACCATCATGATGATGTCAATCAACCCTAAGACTAATAGTACAAGCATTGTTTCAATTCCACGTGACT
CAAATGCAATCTTCCCAGATTACCCACAATATGGTGTAACCAAGATCAACTCAGCTTATACATTGGGTGGAGTTAGT
GAAACTATTAAGACACTTGATAAATATTACAGTGTTCCAATTGACGGCTACATCATGATCAATATGGGTGGTTTGAA
GAAGGCAATTGACCAAGTTGGGGGGATTGATGTAACTTCTCCATTAACTTTCAACAATATGGGTTATAGCTTTGTCA
AAGGCCAGACTTATCACATGAATGGTAAAAAGGCTTTAGCATTTGCCCAACTTCGTCATGGTGACCCTCGTCAGGAT
TATGGTAGACAAGAGCGTGACCGCTTGGTAATTATGGCCTTGCTTAAGAAGTCTGTTTCACCTTCAACTTTGCTTAA
TTCAAGTTTCTTGAATTCCATTTCTAGCGAGATGCAGACTGACTTGACTATGAATCAAATGTACAAGATTGCTATGG
ATTATAGAAAAGCCACTAACAATCTTTCTCAAGATCATGCTCAGGGTGTAAGTAAAGAAACGCAAAACCCTAAGTTT
GGCACTATGGAAATTGAAGTGGTAAGTAGAGAAGAGCGTCAACGAGTTTCTGATAAGATCAGAACGGCGCTAGAATT
GCCACATGTTAAGGTAGCTGCTAACAAGGCTAGCTATATTATGAATCGTTAA

References cited in the Supporting Information

Abraham, M., Alekseenko, A., Bergh, C., Blau, C., Briand, E., Doijade, M., et al. (2023) "GROMACS 2023 Manual." Accessed January 22, 2026. https://zenodo.org/records/7588711

Abraham, M.J., Murtola, T., Schulz, R., Páll, S., Smith, J.C., Hess, B., and Lindahl, E. (2015) GROMACS: High Performance Molecular Simulations Through Multi-Level Parallelism From Laptops to Supercomputers. SoftwareX 1–2: 19–25.

Abramson, J., Adler, J., Dunger, J., Evans, R., Green, T., Pritzel, A., Ronneberger O., Willmore L., Ballard A.J., Bambrick J., Bodenstein S.W., Evans D.A., Hung C.C., O'Neill M., Reiman D., Tunyasuvunakool K., Wu Z., Žemgulytė A., Arvaniti E., Beattie C., Bertolli O., Bridgland A., Cherepanov A., Congreve M., Cowen-Rivers A.I., Cowie A., Figurnov M., Fuchs F.B., Gladman H., Jain R., Khan Y.A., Low C.M.R., Perlin K., Potapenko A., Savy P., Singh S., Stecula A., Thillaisundaram A., Tong C., Yakneen S., Zhong E.D., Zielinski M., Žídek A., Bapst V., Kohli P., Jaderberg M., Hassabis D., Jumper J.M. (2024) Accurate Structure Prediction of Biomolecular Interactions With AlphaFold 3. Nature 630: 493–500.

Arts, R., Zalmstra, B., Vollprecht, W., Jager, T. de, Morcotilo, N., and Hofer, J. (2026) "pixi." Accessed February 18, 2026. https://github.com/prefix-dev/pixi/releases/tag/v0.63.2.

Boonstra, S., Onck, P.R., and Giessen, E. van der (2016) CHARMM TIP3P Water Model Suppresses Peptide Folding by Solvating the Unfolded State. Journal of Physical Chemistry B 120: 3692–3698.

Bouras, G., Judd, L.M., Edwards, R.A., Vreugde, S., Stinear, T.P., and Wick, R.R. (2024) How Low Can You Go? Short-Read Polishing of Oxford Nanopore Bacterial Genome Assemblies. Microbial Genomics 10: 001254.

Bussi, G., Donadio, D., and Parrinello, M. (2007) Canonical Sampling Through Velocity Rescaling. Journal of Chemical Physics 126: 014101.

Charnock, S.J., and Davies, G.J. (1999) Structure of the Nucleotide-Diphospho-Sugar Transferase, SpsA From Bacillus subtilis, in Native and Nucleotide-Complexed Forms,. Biochemistry 38: 6380–6385.

Croatti, V., Dricot, C., Eilers, T., Dillen, J., Van Rillaer, T., Cauwenberghs, E., et al. (2026) Exopolysaccharides of Lactobacillus crispatus Mediate Key Balancing Interactions With the Vaginal Mucosa. npj Biofilms and Microbiomes 12: 70.

Darden, T., York, D., and Pedersen, L. (1993) Particle Mesh Ewald: An N·Log(N) Method for Ewald Sums in Large Systems. Journal of Chemical Physics 98: 10089–10092.

Egorov, A.A., and Atkinson, G.C. (2025) LoVis4u: A Locus Visualization Tool for Comparative Genomics and Coverage Profiles. NAR Genomics and Bioinformatics 7: lqaf009.

Hess, B., Bekker, H., Berendsen, H.J.C., and Fraaije, J.G.E.M. (1997) LINCS: A Linear Constraint Solver for Molecular Simulations. Journal of Computational Chemistry 18: 1463–1472.

Huang, J., Rauscher, S., Nawrocki, G., Ran, T., Feig, M., Groot, B.L., et al. (2017) CHARMM36m: An Improved Force Field for Folded and Intrinsically Disordered Proteins. Nature Methods 14: 71–73.

Huey, R., Morris, G.M., Olson, A.J., and Goodsell, D.S. (2007) A Semiempirical Free Energy Force Field With Charge-Based Desolvation. Journal of Computational Chemistry 28: 1145–1152.

Jorgensen, W.L., Chandrasekhar, J., Madura, J.D., Impey, R.W., and Klein, M.L. (1983) Comparison of Simple Potential Functions for Simulating Liquid Water. The Journal of Chemical Physics 79: 926– 935.

Kassambara, A. (2025) “ggpubr: “ggplot2” Based Publication Ready Plots.” https://cran.r-project.org/web/packages/ggpubr/index.html

Katoh, K., Rozewicki, J., and Yamada, K.D. (2019) MAFFT Online Service: Multiple Sequence Alignment, Interactive Sequence Choice and Visualization. Briefings in Bioinformatics 20: 1160–1166.

Koes, D.R., Baumgartner, M.P., and Camacho, C.J. (2013) Lessons Learned in Empirical Scoring With Smina From the CSAR 2011 Benchmarking Exercise. Journal of Chemical Information and Modeling 53: 1893–1904.

Kolmogorov, M., Yuan, J., Lin, Y., and Pevzner, P.A. (2019) Assembly of Long, Error-Prone Reads Using Repeat Graphs. Nature Biotechnology 37: 540–546.

Kuraku, S., Zmasek, C.M., Nishimura, O., and Katoh, K. (2013) aLeaves Facilitates On-Demand Exploration of Metazoan Gene Family Trees on MAFFT Sequence Alignment Server With Enhanced Interactivity. Nucleic Acids Research 41: W22–W28.

Le Guilloux, V., Schmidtke, P., and Tuffery, P. (2009) Fpocket: An Open Source Platform for Ligand Pocket Detection. BMC Bioinformatics 10: 168.

Li, H. (2016) Minimap and Miniasm: Fast Mapping and de Novo Assembly for Noisy Long Sequences. Bioinformatics 32: 2103–2110.

McGibbon, R.T., Beauchamp, K.A., Harrigan, M.P., Klein, C., Swails, J.M., Hernández, C.X., Schwantes C.R., Wang L.P., Lane T.J., Pande V.S. (2015) MDTraj: A Modern Open Library for the Analysis of Molecular Dynamics Trajectories. Biophysical Journal 109: 1528–1532.

Nguyen, H., Case, D.A., and Rose, A.S. (2018) NGLview–Interactive Molecular Graphics for Jupyter Notebooks. Bioinformatics 34: 1241–1242.

Nosé, S., and Klein, M.L. (1983) Constant Pressure Molecular Dynamics for Molecular Systems. Molecular Physics 50: 1055–1076.

Oxford Nanopore Technologies (2018) “medaka.” Accessed October 12, 2024. https://github.com/nanoporetech/medaka

Páll, S., and Hess, B. (2013) A Flexible Algorithm for Calculating Pair Interactions on SIMD Architectures. Computer Physics Communications 184: 2641–2650.

Parks, D.H., Imelfort, M., Skennerton, C.T., Hugenholtz, P., and Tyson, G.W. (2015) CheckM: Assessing the Quality of Microbial Genomes Recovered From Isolates, Single Cells, and Metagenomes. Genome Research 25: 1043–1055.

Parrinello, M., and Rahman, A. (1981) Polymorphic Transitions in Single Crystals: A New Molecular Dynamics Method. Journal of Applied Physics 52: 7182–7190.

R Core Team (2021) “R: A Language and Environment for Statistical Computing.”. https://www.R-project.org/.

Sadovskaya, I., and Guérardel, Y. (2019) Simple Protocol to Purify Cell Wall Polysaccharide From Gram-Positive Bacteria and Assess Its Structural Integrity. In Bacterial Polysaccharides: Methods and Protocols. Brockhausen, I. (ed.). Springer, New York, NY. pp. 37–45 . Accessed August 1, 2025. 10.1007/978-1-4939-9154-9_4.

Schneider, C.A., Rasband, W.S., and Eliceiri, K.W. (2012) NIH Image to ImageJ: 25 Years of Image Analysis. Nature Methods 9: 671–675.

Schwengers, O., Jelonek, L., Dieckmann, M.A., Beyvers, S., Blom, J., and Goesmann, A. (2021) Bakta: Rapid and Standardized Annotation of Bacterial Genomes via Alignment-Free Sequence Identification. Microbial Genomics 7: 000685.

Tarbouriech, N., Charnock, S.J., and Davies, G.J. (2001) Three-Dimensional Structures of the Mn and Mg dTDP Complexes of the Family GT-2 Glycosyltransferase SpsA: A Comparison With Related NDP-Sugar Glycosyltransferases 1. Journal of Molecular Biology 314: 655–661.

Van der Veer, C., Hertzberger, R.Y., Bruisten, S.M., Tytgat, H.L.P., Swanenburg, J., De Kat Angelino-Bart, A., et al. (2019) Comparative Genomics of Human Lactobacillus crispatus Isolates Reveals Genes for Glycosylation and Glycogen Degradation: Implications for in Vivo Dominance of the Vaginal Microbiota. Microbiome 7: 49.

Vaser, R., and Šikić, M. (2021) “Raven: A De Novo Genome Assembler for Long Reads.”. 2020.08.07.242461. Accessed November 28, 2025. 10.1101/2020.08.07.242461v2.

Wang, J.-C., Lin, J.-H., Chen, C.-M., Perryman, A.L., and Olson, A.J. (2011) Robust Scoring Functions for Protein–Ligand Interactions With Quantum Chemical Charge Models. Journal of Chemical Information and Modeling 51: 2528–2537.

Wick, R.R., and Holt, K.E. (2022) Polypolish: Short-Read Polishing of Long-Read Bacterial Genome Assemblies. PLoS Computational Biology 18: e1009802.

Wick, R.R., Judd, L.M., Cerdeira, L.T., Hawkey, J., Méric, G., Vezina, B., Wyres K.L., Holt K.E. (2021) Trycycler: Consensus Long-Read Assemblies for Bacterial Genomes. Genome Biology 22: 266.

Wick, R.R., Schultz, M.B., Zobel, J., and Holt, K.E. (2015) Bandage: Interactive Visualization of de Novo Genome Assemblies. Bioinformatics 31: 3350–3352.

Wickham, H., Bryan, J., Posit, attribution P. (Copyright holder of all R. code and all C. code without explicit copyright, code), M.K. (Author of included R., code), K.V. (Author of included libxls), et al. (2025a) "readxl: Read Excel Files." Accessed November 28, 2025. https://cran.r-project.org/web/packages/readxl/index.html

Wickham, H., Chang, W., Henry, L., Pedersen, T.L., Takahashi, K., Wilke, C., et al. (2025b) "ggplot2: Create Elegant Data Visualisations Using the Grammar of Graphics." Accessed November 28, 2025. https://cran.r-project.org/web/packages/ggplot2/index.html

Wickham, H., and RStudio (2023) "tidyverse: Easily Install and Load the Tidyverse." Accessed November 28, 2025. https://cran.r-project.org/web/packages/tidyverse/index.html

Wickham, H., Vaughan, D., Girlich, M., Ushey, K., Software, P, and PBC (2024) "tidyr: Tidy Messy Data." Accessed November 28, 2025. https://cran.r-project.org/web/packages/tidyr/index.html
